# Infrared Spectroscopy and Photochemistry of Anthracoronene in Cosmic Water Ice

**DOI:** 10.1021/acsearthspacechem.1c00337

**Published:** 2022-01-09

**Authors:** Julie M. Korsmeyer, Alessandra Ricca, Gustavo A. Cruz-Diaz, Joseph E. Roser, Andrew L. Mattioda

**Affiliations:** † NASA Ames Research Center, Mail Stop 245-6, Moffett Field, California 94035-1000, United States; ‡ Department of Chemistry, University of Chicago, 5735 S. Ellis Avenue, Chicago, Illinois 60627 United States; § Carl Sagan Center, SETI Institute, 189 Bernardo Avenue, Mountain View, California 94043, United States; ∥ BAER Institute, P.O. Box 25, Moffett Field, California 94035-1000, United States

**Keywords:** astrochemistry, molecular spectroscopy, ISM, PAHs, water ice

## Abstract

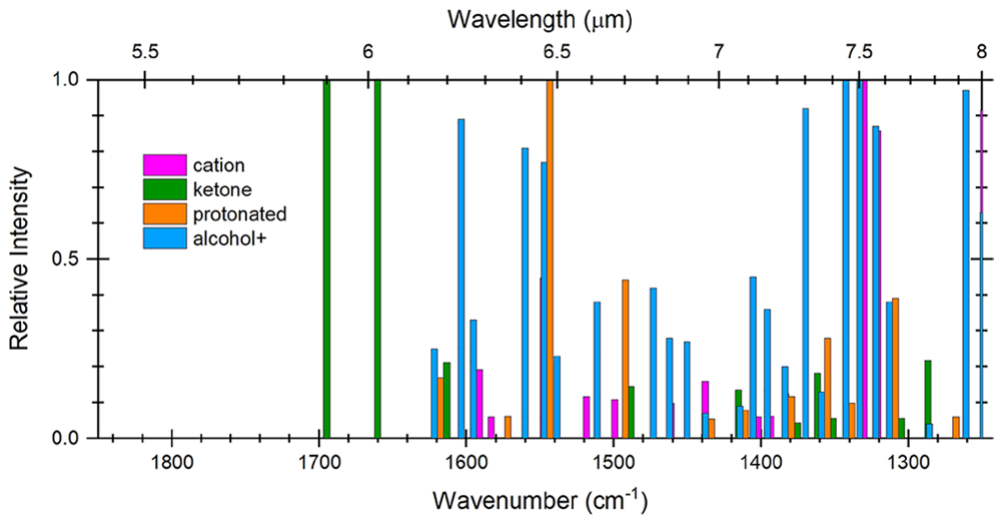

We present a laboratory study of the polycyclic aromatic hydrocarbon (PAH) anthracoronene (AntCor, C_36_H_18_) in simulated interstellar ices in order to determine its possible contribution to the broad infrared absorption bands in the 5–8 μm wavelength interval. The Fourier transform infrared (FTIR) spectrum of AntCor, codeposited with water ice, was collected. The FTIR spectrum of the sample irradiated with ultraviolet photons was also collected. Unirradiated and UV-irradiated AntCor embedded in water ice have not been studied before; therefore, the molecule’s band positions and intensities were compared to published data on AntCor in an argon matrix and theoretical calculations (DFT), as well as the published results of its parent molecules, coronene and anthracene, in water ice. The experimental band strengths for unirradiated AntCor exhibit variability as a function of PAH:H_2_O concentration, with two distinct groupings of band intensities. AntCor clustering occurs for all concentrations and has a significant effect on PAH degradation rates and photoproduct variability. Near-IR spectra of irradiated AntCor samples show that AntCor^+^ production increases as the concentration of AntCor in water ice decreases. Photoproduct bands are assigned to AntCor^+^, cationic alcohols, protonated AntCor, and ketones. We report the rate constants of the photoproduct production for the 1:1280 AntCor:H_2_O concentration. CO_2_ production from AntCor is much less than what was previously reported for Ant and Cor and exhibits two distinct regimes as a function of AntCor:H_2_O concentration. The contribution of AntCor photoproducts to astronomical spectra can be estimated by comparison with the observed intensities in the 7.4–8.0 μm range.

## 1 Introduction

In dense molecular clouds, comets, the ice mantles on interplanetary dust particles (IDPs), and many Solar System objects, polycyclic aromatic hydrocarbons (PAHs) can be incorporated into H_2_O ice.^1^ Indeed, the presence of PAHs in the dense clouds associated with young stellar objects (YSO’s) has been suggested by infrared absorption bands observed in the 5–8 μm spectral region, which are indicative of aromatic C–C stretching and C–H in-plane bending modes.^2−6^ In some instances, the 3.25 μm C–H aromatic stretching band was detected in these objects as well.^7−10^ Upper limits for the abundance of PAHs in ices ranging from 2–3%^11^ up to 12%^12^ have been reported. Large PAHs (i.e., >24 carbon atoms) are abundant in the ISM^13^ and can be incorporated into water ices, contributing to the absorption at 3.25 μm,^12^ yet very little laboratory data are available for PAHs of this size.^14,15^ In addition, previous laboratory work has focused on either pericondensed or catacondensed PAHs. Here we investigate the infrared spectroscopy and photochemistry of anthracoronene (AntCor, C_36_H_18_) in water ices. AntCor is a unique, large PAH with a structure obtained by the fusion of the pericondensed coronene and catacondensed anthracene subunits. We study the effect of AntCor concentration in amorphous H_2_O ices on the MIR spectra of unirradiated AntCor and on its photochemistry and compare the results to those previously obtained for anthracene and coronene in H_2_O ices.^16−25^ Thus, this study investigates if the previously observed trends are valid for mixed pericondsed/catacondensed PAHs containing more than 24 carbon atoms.

This paper is organized as follows: Section 2 describes the experimental and theoretical procedures used in the study. In Section 3 spectral data for unirradiated AntCor as a function of concentration are compared with spectral data of anthracene and coronene. Section 4 discusses the photochemistry of AntCor, the effect of concentration on the formation of photoproducts, and the identification of the photoproducts. Near-IR spectra are also presented for various concentrations. The astrophysical implications are discussed in Section 5, and the conclusions are given in Section 6.

## 2 Methodologies

### 2.1 Experimental Setup

**Figure 1:**
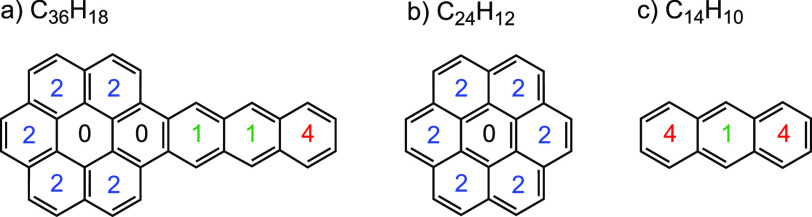
Molecular structures of (a) anthracoronene (AntCor, C_36_H_18_), (b) coronene (Cor, C_24_H_12_), and (c) anthracene (Ant, C_14_H_10_). Rings are labeled with the number of vicinal hydrogens.

In this work, the mid-infrared (MIR, 4000–500 cm^–1^, 1.67–25 μm) spectra of unirradiated and UV-irradiated anthracoronene (AntCor, C_36_H_18_) and the near-infrared (NIR, 15000–7000 cm^–1^, 0.66–1.43 μm) spectra of UV-irradiated AntCor embedded in an amorphous water ice matrix are reported for the first time. The structures of AntCor, coronene, and anthracene are shown in Figure 1. No unexpected safety hazards were encountered during the experiments.

**Figure 2:**
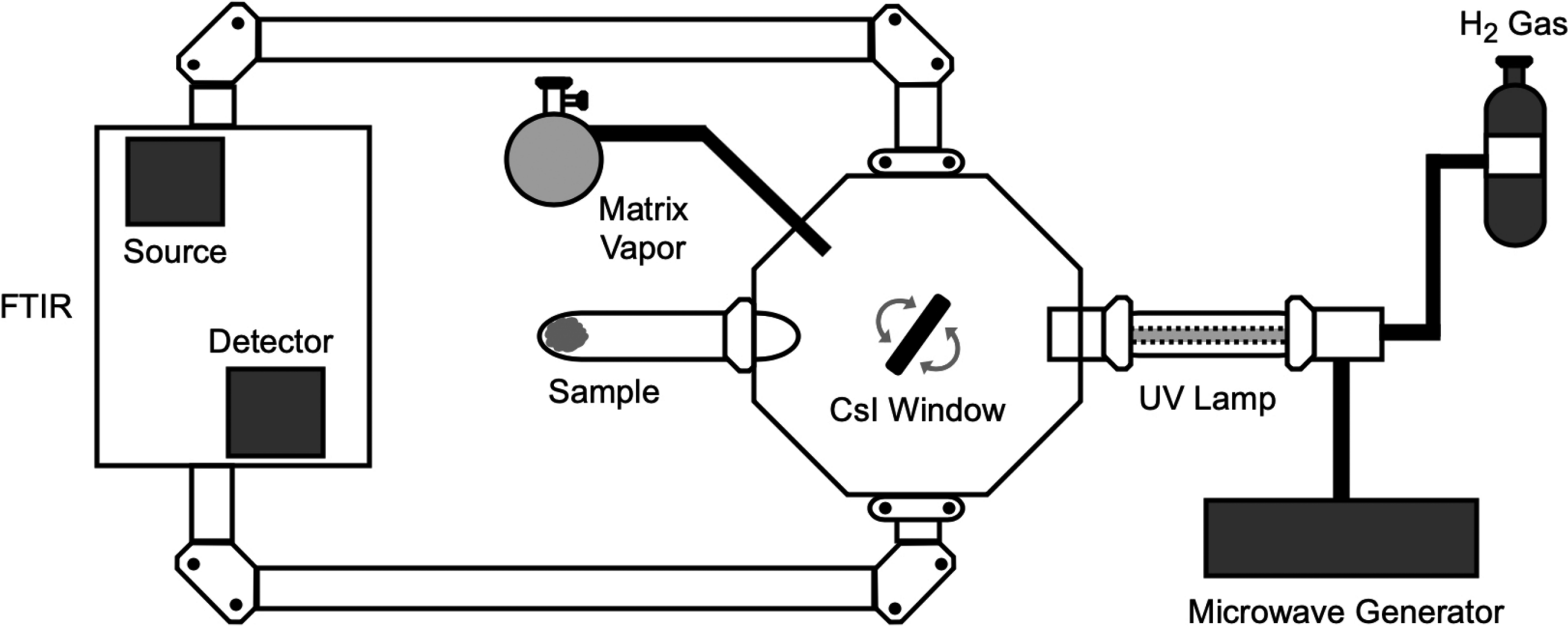
Schematic of the experimental setup. The window in the main chamber can rotate 360° to face the appropriate inlet(s) for deposition, irradiation, and spectroscopy.

Amorphous ice samples were prepared by the vapor codeposition of AntCor (anthra[2,3-*a*]coronene, 99%, Chiron, CAS 5869-17-0) and H_2_O onto an IR-transparent, cryogenically cooled, CsI window suspended in a high-vacuum chamber (*P* ≈ 10^–8^ Torr) as shown in Figure 2. The CsI window was held at a temperature of 18 K throughout the deposition and experiment. Deposition of the AntCor was accomplished by hermetically sealing a Pyrex tube containing AntCor to the vacuum chamber. The tube was heated to 360 °C, AntCor’s sublimation temperature,^43^ vaporizing the PAH into the sample chamber. Simultaneously, water vapor was deposited through an adjacent port, producing an AntCor:H_2_O ice layer on the CsI window. For the deposition process, the CsI window was rotated to face exactly between the Pyrex tube and matrix vapor inlets, 22.5° from each inlet, as shown in Figure 2. The water used was deionized, Milli-Q grade filtered water, purified of contaminant gases by at least three freeze–pump–thaw cycles performed on a Schlenk line. To prepare the different levels of AntCor:H_2_O concentrations (1:40–1:1280), the water flow was varied while all other parameters were held constant. The duration of deposition was tuned to obtain samples of similar thicknesses across experiments.

After AntCor:H_2_O deposition was completed, a MIR spectrum of the unirradiated (i.e., nonirradiated) ice sample was collected using a Biorad Excalibur FTS 4000 FTIR spectrometer with a resolution of 0.5 cm^–1^. NIR spectra were collected with a resolution of 1 cm^–1^. MIR spectra were collected using a KBr broad-band beam splitter and a liquid-nitrogen-cooled MCT-B detector. The 12000–7000 cm^–1^ NIR spectral region required the use of a silicon detector in conjunction with a quartz beam splitter and tungsten lamp.

After the spectrum of the unirradiated ice was collected, the sample was photolyzed with ultraviolet (UV) radiation using a flowing-H_2_ microwave discharge lamp set to a dynamic pressure of 1.5 × 10^–1^ Torr. In order to mimic interstellar conditions as accurately as possible, the spectrum from the lamp includes the combined 121.6 nm Lyman α (10.2 eV) and 160 nm (7.8 eV) molecular hydrogen emission bands. The UV radiation from the lamp enters the sample chamber through an adjacent MgF_2_ window. The photon flux of the lamp, calculated by the actinometrical measurement method,^26,27^ was (2 ± 0.5) × 10^14^ photons/cm^2^s. The UV lamp was maintained at a forward power of 100 W and a reflected power of <2 W. MIR spectra of the sample ice were collected after 0, 2, 4, 8, 16, 32, 64, and 128 min of radiation exposure. NIR spectra were taken after deposition and after 128 min of VUV irradiation. MIR and NIR single-beam spectra were processed against their respective backgrounds. No unexpected safety hazards were encountered during the experiments.

Weaker photoproduct bands are difficult to identify in the raw irradiated spectra due to the production of multiple new bands and spectral noise. In order to isolate the photoproduct bands from the neutral AntCor bands, the spectrum of the unirradiated sample was subtracted from the spectrum of the irradiated sample. In a similar fashion, a spectrum of air was subtracted off to remove interference from atmospheric bands. All baseline corrections, spectral subtractions, and filtering were performed using BioRad’s Agilent Resolutions Pro software, version 4.0.11. All tabulated and calculated results presented here were determined from unfiltered, nonbaselined data.

### 2.2 Theoretical Calculations

**Figure 3:**
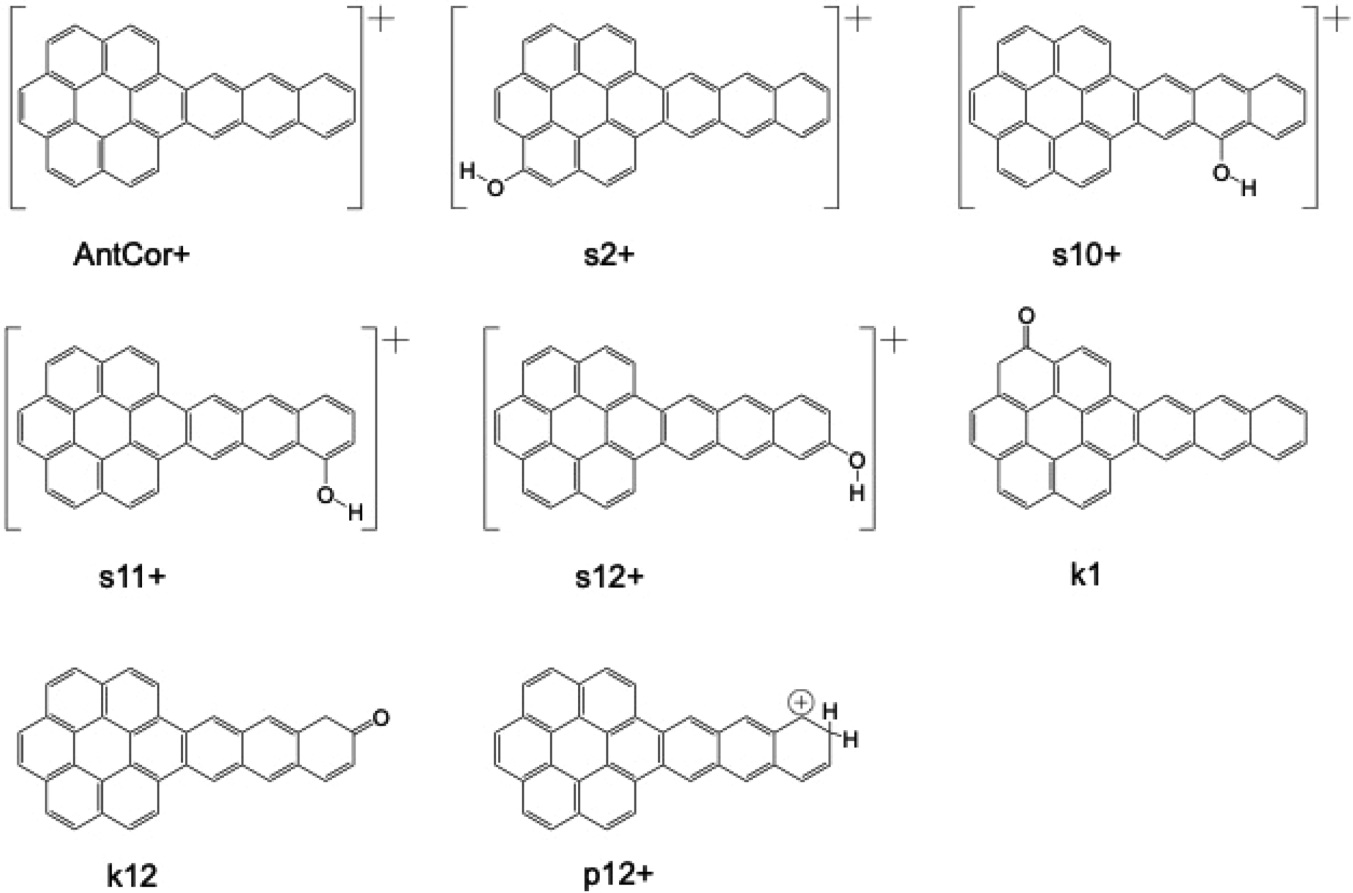
AntCor:H_2_O photoproduct structures, identified by a comparison between experimental and theoretical MIR spectra, are AntCor^+^, cationic aromatic alcohols (AntCorOH^+^: s2+, s10+, s11+, s12+), ketones (AntCorO: k1, k12), and protonated AntCor (AntCorH^+^: p12+).

We have considered four classes of neutral and ionized AntCor derivatives, namely alcohols, ketones, quinones, and protonated AntCor, as suggested by previous photochemical studies.^20−22^ The short list of AntCor:H_2_O photoproducts, shown in Figure 3, was obtained by a comparison with experimental data. The geometries of all the structures shown in Figure 3 were fully optimized and the harmonic frequencies computed using density functional theory (DFT). We used the hybrid B3LYP functional,^28,29^ in conjunction with the cc-pVTZ basis set.^30^ In calculations of the AntCor dimer, the dispersion effects were described using the D3 version of dispersion and the D3 damping function from a previous study.^31^ All of the calculations were performed using the Gaussian09 suite of programs.^32^ The computed harmonic frequencies were scaled to lower frequencies using three scale factors: namely, 0.964 for C–H stretches, 0.979 for the 4–9 μm region, and 0.975 for the region >9 μm. The scale factors were obtained by fitting to 25 bands obtained from gas-phase PAH experiments, which include 17 infrared-allowed bands and 1 A_u_, 1 B_1g_, 2 B_2g_, and 4 A_g_ bands.^33−35^ The integrated band intensities were broadened by 7 cm^–1^ to produce synthetic spectra that were comparable with experimental band widths. The calculations did not include overtones, combination bands, and resonances, which are expected to be weak in comparison with the fundamentals. The molecular graphics tool Jmol was used to visualize the molecular vibrations.^36^

### 2.3 Calculated Band Strength

The integrated absorbances of the measured AntCor bands were determined from the sum of all theoretical band intensities between 1550 and 1000 cm^–1^ (6.5 and 10.0 μm, respectively). These spectral limits were chosen to exclude the contributions of far-IR bands (<500 cm^–1^), C–H stretching bands (>3000 cm^–1^), and interference from the H_2_O libration (850–750 cm^–1^; 11.77–13.33 μm) and stretching (1700–1580 cm^–1^; 6.33–5.88 μm) modes. An advantage of this method is that even though there may be some band to band variability in the accuracy of the calculated intensity, the total intensity sum is generally accurate to within 10–20%.^37^

To determine the experimental integrated band strength of an AntCor mode in water, we first characterized the number of molecules present in the sample in terms of column density, *N*. All bands present between 1550 and 1000 cm^–1^ in a spectrum of unirradiated ice had their band areas integrated and then summed. The same was done for all DFT-calculated modes in that region. *N* is the column density (molecules/cm^2^), determined by the ratio of the sum of experimental modes to the sum of theoretical modes within the same region. The experimental band strength, *A*, of an AntCor mode is found by scaling the experimental integrated band area using the *N* value. A band’s area is multiplied by a factor of 2.303 to convert the absorption from log_10_ to a natural log (ln). That corrected band area is then divided by *N* to get the value of *A* (cm/molecule; see eq 1).
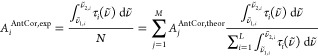
1τ_*i*_(ν) is the optical depth of mode *i* in H_2_O ice at wavenumber ν̃ (cm^–1^), A_*j*_^AntCor,theor^ (cm/molecule) is the theoretically calculated absolute intensity of vibrational mode *j* in the 1600–1000 cm^–1^ range, *M* is the number of theoretically calculated modes in the same region, *L* is the number of measured modes, and ν̃_1,*i*_ and ν̃_2,*i*_ are respectively the lower and upper integration boundaries in cm^–1^ for absorption feature *i*.

To determine the concentration of AntCor:H_2_O in the sample, the column density of water is calculated by following the same steps in eq 1 using literature values rather than theoretical intensities: 2.8 × 10^–17^ cm/molecule for the 760 cm^–1^ water band, 1.0 × 10^–17^ cm/molecule for 1657 cm^–1^, and 1.7 × 10^–16^ cm/molecule for 3298 cm^–1^.^38^ Using the ratio of column densities between water and AntCor, we determined AntCor:H_2_O concentrations of 1:40 (±5), 1:60 (±10), 1:130 (±15), 1:390 (±50), 1:420 (±60), 1:590 (±80), and 1:1280 (±150), which allowed us to study in a systematic way the effect of PAH concentration on the photochemistry of AntCor in H_2_O ices. This calculation for *N* does not take solvation and clustering into account.^39^

### 2.4 Computed PAH Monomer Fractions

To compute the monomer fractions, we used the model described in a previous work,^40^ where each PAH molecule is represented by the union of sphere-swept disks and each disk represents one of the aromatic rings in the PAH. The monomer fraction was estimated using the following three steps. First, the modeled molecules were randomly distributed, with a random orientation, within a cubic space. Then, the model applied a global mark process^41^ to resolve overlapping molecules. Finally, the interior volume of the trial space was used to calculate the volume fraction occupied by the remaining PAH molecules and the monomer fraction using a nearest-neighbor threshold distance of 5 Å.^21^

To connect experimental measurements to monomer fractions, the empty volume fraction *E* for each experimental measurement was estimated using eq 2
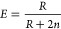
2where *R* is the ratio of water molecules to embedded PAH molecules in a measurement and 2*n* is the number of water molecules assumed to be displaced by a PAH molecules with *n* rings (i.e., 2*n* = 20 for AntCor). The approximation 1 – *E* = 2*n*/*R* represents the *R* ≫ 2*n* limit of this equation, which is the regime in which matrix isolation experiments on PAHs are generally conducted.^42^ While this slightly overestimates the volume of an AntCor molecule, it provides a reasonable estimate for the monomer fraction calculation. The monomer fractions for the experimental matrix deposits were then determined by fitting a quadratic curve to the computed monomer fraction as a function *E* and then interpolating.

The monomer fraction for the different AntCor:H_2_O concentrations were calculated to be 50% for the 1:1280 ratio, 20% for the 1:590 ratio, 9% for the 1:420 ratio, 8% for the 1:390 ratio, and 0% for the remaining concentrations. Thus, even at the lowest PAH concentrations studied here, the monomer fraction constitutes only 50% of the sample. The AntCor monomer fraction is very small for moderate to high PAH concentration (i.e., 1:390, 1:420) which affects the ionization fraction and the photochemistry of the ice sample. Using the same model, the monomer fractions for AntCor in Ar at the same concentrations are 62% for the 1:1280 ratio, 34% for the 1:590 ratio, 21% for the 1:420 ratio, 18% for the 1:390 ratio, and 0% for the remaining concentrations. The monomer fractions are smaller in water ice than in Ar ice, as the volume occupied by an Ar atom is larger than the volume occupied by an H_2_O molecule. AntCor has smaller monomer fractions in both water and Ar ice than Ant and Cor at similar concentrations, due to the difference in PAH sizes. For example, at a concentration of 1:1280 PAH:H_2_O, the monomer fraction is 77% for Ant and 64% for Cor in compariosn to 50% for AntCor.

## 3 Unirradiated AntCor in Water Ice

This section discusses the experimental data for MIR band positions and intensities. Previous studies were used to identify modes of interest in comparison to AntCor’s component molecules, anthracene (Ant) and coronene (Cor).^1,20−22,43^ We also discuss the differences in the intensity and position of unirradiated AntCor bands in an Ar matrix versus water ice. This discussion focuses on 1700–1000 cm^–1^, with the 1700–1500 and 1000–700 cm^–1^ regions having greater uncertainty due to overlap with water bands. The full MIR spectra for unirradiated AntCor:H_2_O at each concentration are provided in Figures S1 and S2 in the Supporting Information.

### 3.1 Comparison to Unirradiated Cor:H_2_O and Ant:H_2_O

As previously stated, AntCor can be considered a combination of the coronene (Cor) and anthracene (Ant) molecules. It is therefore interesting to compare our experimental results with the data of the two “parent” molecules to see if AntCor behaves more like one of its substructures than the other.

The structure of AntCor contains one quartet, five duo, and four solo sets of vicinal hydrogens (Figure 1). Coronene has a highly symmetric structure with D_6*h*_ symmetry and contains only dual vicinal hydrogens on all of its outer rings. Anthracene has *D*_2*h*_ symmetry and consists of three fused benzene rings arranged linearly with rows of aligned C–H bonds: quartet hydrogens on the end rings and a solo hydrogen on the central ring. In AntCor, the Ant and Cor units have *C*_2*v*_ symmetry and previously inactive IR bands become active.

The experimental data on unirradiated anthracene and coronene in water ice were reported in previous studies.^20,22^Table 1 shows a comparison among AntCor, Cor, and Ant, all in water ice, in the MIR region and at similar PAH:H_2_O concentrations to avoid any discrepancies coming from significant concentration variance.

**Table 1:** Comparison of Experimental In- and Out-of-Plane Band Positions (cm^–1^) for Unirradiated AntCor (1:130), Coronene (1:150), and Anthracene (1:60) in Water Ices with Their Respective *A* (10^–19^ cm/molecule) Values^a^

AntCor:H_2_O (1:130)	*A*	mode	Cor:H_2_O (1:150)^22^	*A*	Ant:H_2_O (1:60)^20^	*A*
1003.0	7.3	Ant			1001.7	6.8
1017.6	12.5	Cor				
					1103.8	0.8
1124.2	7.1	Ant			1127.4	1.8
1133.7	13.1	Cor	1137.3	21.3		
1151.0	11.5	Cor			1149.7	9.0
					1168.2	6.1
					1186.8	1.0
1221.2	9.6	Cor	1212.6	1.8		
1262.1	12.8	Cor				
1282.6	7.3	Ant			1272.3	5.6
1296.7	13.1	Ant				
1318.4	11.5	Cor	1317.3	76.4	1316.0	10.5
					1328.3	2.2
1340.8	30.4	Ant, Cor			1347.7	3.5
1370.2	6.0	Ant				
1405.4	10.1	Ant			1400.1	3.2
1420.1	10.3	Ant				
1438.7	14.8	Ant			1450.9	14.6
1482.8	6.6	Cor				
			1498.9	2.5		
1513.5	5.6	Cor				
			1532.4	6.1	1537.9	8.6
					1563.1	0.8
1610.2	7.0	Cor	1603.0	41.0		
			1618.9	9.6	1624.4	8.0

a AntCor:H_2_O bands with *A* < 5 × 10^–19^ cm/molecule are not reported. For each AntCor band, it is indicated whether there is a corresponding mode for Ant and/or Cor.

**Figure 4:**
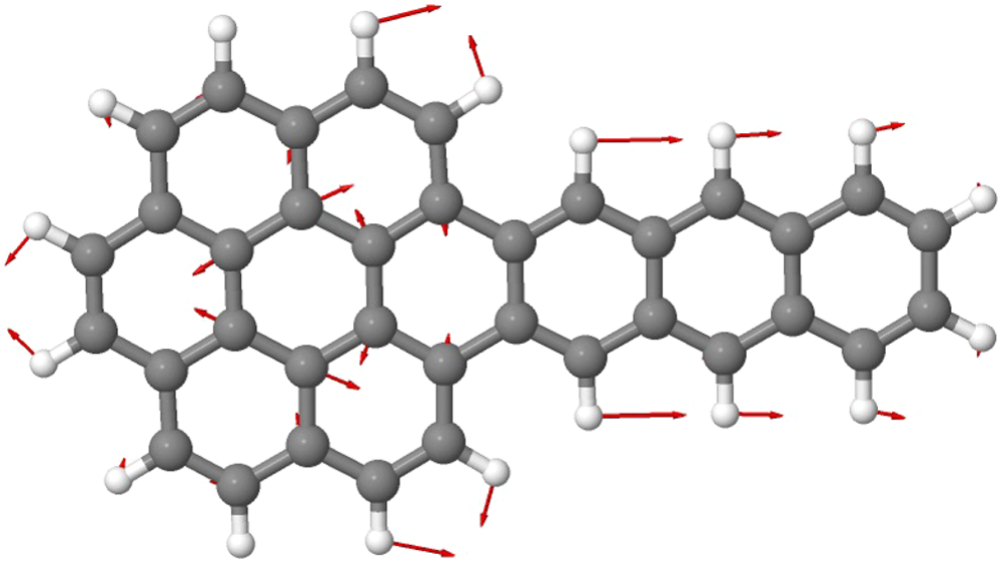
Vibrational mode of unirradiated AntCor in water ice which produces the band at 1341 cm^–1^. The displacement vectors are shown in red. The molecular structure and the vibrational mode were visualized using Jmol.^36^

In the C–C and C–H in-plane region (1000–1500 cm^–1^), 16 AntCor:H_2_O bands were observed (Table 1). The AntCor:H_2_O bands at 1003, 1124, 1283, 1297, 1370, 1405, 1420, and 1439 cm^–1^ have a strong Ant component with several bands appearing at positions similar to those for Ant:H_2_O.^20^ The difference in band positions between the two samples varies by up to 10 cm^–1^, and the *A* values of AntCor:H_2_O (1:130) are overall much greater than those of Ant:H_2_O (1:60). The AntCor:H_2_O bands at 1018, 1134, 1151, 1221, 1262, 1318, 1483, 1514, and 1610 cm^–1^ have a strong Cor component. The modes of AntCor that have vibrational frequencies similar to bands observed in Ant or Cor are identified as the same modes. The remaining band at 1341 cm^–1^ has similar contributions from modes on both Ant and Cor portions, as is shown in Figure 4.

### 3.2 Effects of Concentration on Unirradiated AntCor:H_2_O

**Figure 5:**
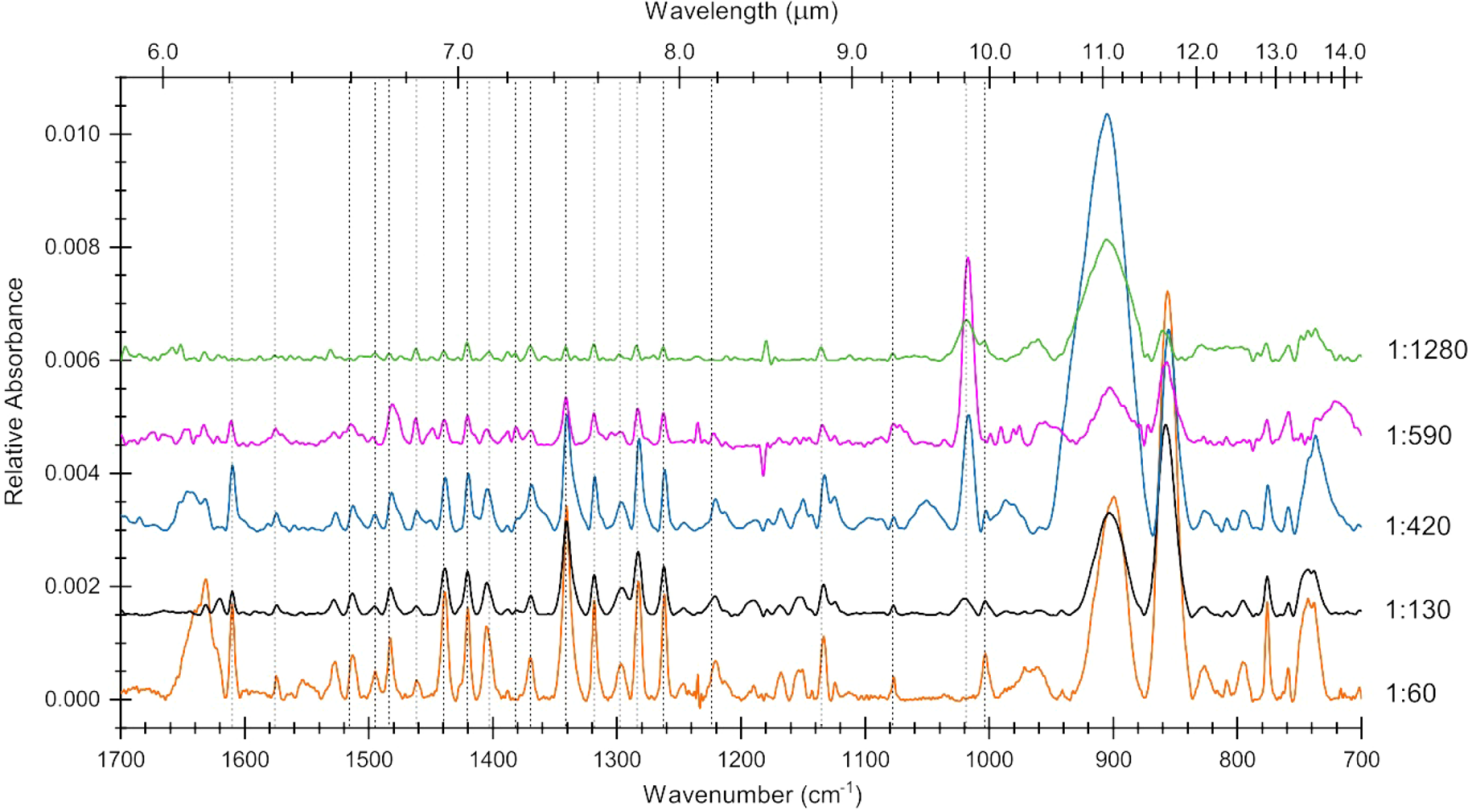
Mid-IR spectra from 1700 to 700 cm^–1^ of unirradiated AntCor:H_2_O showing the relative intensities of bands across concentrations of 1:60 (green), 1:130 (black), 1:420 (blue), 1:590 (magenta), and 1:1280 (green). Vertical dashed lines indicate the center of unirradiated bands in the 1:1280 concentration, to demonstrate shifting as PAH clustering increases. The spectra are artificially vertically offset for clearer presentation. Each spectrum has been baseline-corrected and filtered.

Figure 5 displays spectra of unirradiated AntCor:H_2_O samples for five concentrations. The figure shows how the band intensities of unirradiated AntCor decrease as the AntCor concentration is reduced, such as those of the 1260, 1400, and 1610 cm^–1^ bands. The few negative peaks appearing at around 1190 and 1215 cm^–1^ are residual artifacts from the background spectra and do not represent any real peaks.

**Figure 6:**
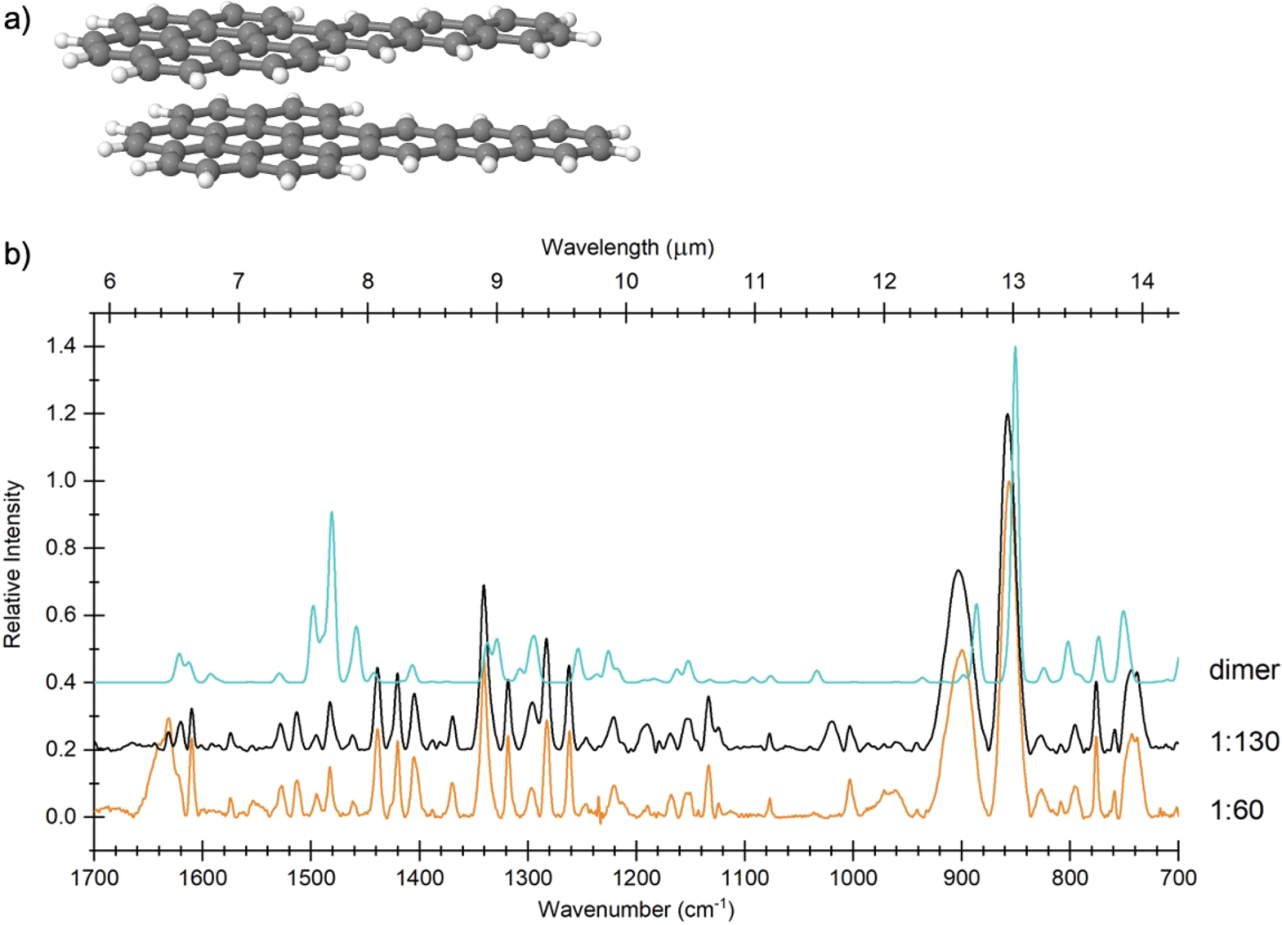
(a) Computed structure of the AntCor dimer visualized using Jmol.^36^ (b) Mid-IR spectra from 1700 to 700 cm^–1^ of unirradiated AntCor:H_2_O showing the relative intensities of bands at concentrations of 1:60 (orange) and 1:130 (black) and the computed spectrum of the AntCor dimer (cyan). All spectra have been normalized to their most intense band, and the experimental spectra were baseline-corrected and filtered.

The spectra of unirradiated AntCor:H_2_O at concentrations of 1:60 and 1:130 have profiles that are comparable to those of the calculated spectrum for the AntCor dimer (Figure 6). The dimer displayed in Figure 6 is the lowest energy configuration; however, given that the ices are formed at 18 K, other configurations might be present as well since not enough thermal energy is available for molecules to rearrange. The 1610 cm^–1^ C–C stretch is quite sharp for the 1:60 experiment but smaller for the 1:130 concentration, implying that clustering has a significant effect on this vibrational mode at high PAH concentrations. The vertical dotted lines in Figure 5 show how some bands exhibit slight shifts in position due to clustering at high PAH concentrations, in agreement with previous studies.^42,44^

Some bands, like those at 1631, 898, 855, and 743 cm^–1^, show changes in their relative intensities between high and low PAH concentrations (Figure 6). The band at 1631 cm^–1^ is due to the C=C stretching modes of the aromatic rings, the 898 cm^–1^ band is due to “solo” out-of-plane C–H bending modes (CH_oop_) modes on the Ant portion of AntCor, and the 732 cm^–1^ band is due to “quartet” CH_oop_ modes also on the Ant portion, while the 855 cm^–1^ band is due to “duo” CH_oop_ modes on the Cor portion. Figure 5 shows that changes in relative band intensities start to occur at an intermediate PAH concentration (i.e., 1:420) and persist through the 1:1280 concentration. This change in relative intensity is due to an increased importance of water solvation and the arrangement of AntCor molecules in the presence of water. Table 2 provides a record of unirradiated AntCor band positions and intensities in water ice, at all studied concentrations. Minor variations between the spectra can be due to small differences in the baseline corrections or filtering. For example, the bands between 1000 and 700 cm^–1^ and between 1700 and 1550 cm^–1^ are at the edge of broad water modes, which causes small distortions to bands when the spectra are baseline-corrected.

**Table 2:** Summary of Average Band Positions and *A* Values of Unirradiated AntCor:H_2_O in the MIR region (1700–500 cm^–1^), at All Concentrations^a^

	AntCor:H_2_O						
position (cm^–1^)	1:40^b^	1:60^c^	1:130^d^	1:390^e^	1:420^f^	1:590^g^	1:1280^h^
543.1	21.3	26.2	21.4	27.4	24.1	16.4	
576.2	37.8	38.5	37.3	31.6	37.9	19.8	53.5
595.1	7.0	9.9					
653.6						16.9	
671.7		7.7					
743.0	48.2	47.6	20.2				
758.9	3.3*	4.1	5.0	5.5*	8.6		
775.8	7.6	9.3	9.2	5.8*	9.0		
794.7	8.7	8.8	12.1				
826.9	11.4	5.9	11.4				
856.5	146.7	120.5	89.1	30.9	99.4	18.7	44.5
898.7	78.3	41.0	26.6		84.4		
1003.0	6.7	8.4	7.3	4.2*	6.3		9.0*
1016.9			12.5	5.5*	40.8	98.3	12.2*
1077.0	2.3*	2.9*	3.0*	5.1*	2.9*		12.7*
1124.4	3.4*	4.0	7.1	4.4*	7.4		
1133.4	8.7	9.8	13.1	12.7	12.4	21.4	14.6*
1143.3	2.5*	2.8*					
1152.4	7.4	9.6	11.5	5.8*	5.6		
1168.6	5.6	4.9	7.5	3.8*	7.5		
1189.8	1.6*	1.3*					
1211.7	2.6*	3.6	2.5*	2.4*			
1221.1	8.5	9.1	9.6	7.0*	6.5	5.1*	12.8*
1246.8	3.3*	2.1*	3.1*	4.0*			
1262.2	11.6	10.8	12.8	13.4	11.4	11.1	14.1*
1283.0	19.6	19.5	7.3	18.1	16.8	12.2	16.8
1296.4	13.1	12.0	13.1	11.6	10.5		10.7*
1318.4	9.3	11.0	11.4	13.3	8.2	6.2*	10.5*
1341.0	34.0	35.4	30.4	43.4	20.6	13.6	21.2
1369.5	4.2	5.7	6.0	6.1*	5.1*		11.5*
1380.0			2.1*				15.8
1388.1	2.1*	0.7*	2.7*	3.0*			
1405.3	13.3	12.8	10.2	11.6	7.2	5.2*	13.2*
1420.3	12.1	9.8	10.3	10.6	11.4	7.7	13.9*
1426.8		1.4*					
1438.7	18.3	15.5	14.8	8.7*	10.4	6.5*	14.3*
1462.3	3.7	2.2*	3.2*	4.8*	3.1*	7.9	13.0
1482.6	5.6	6.1	6.6	11.7	6.7	19.2	11.0*
1494.3	2.8*	3.1	3.2*	6.2*	3.3*	3.3*	5.7*
1513.3	8.3	6.6	5.6	8.0*	7.0	6.0*	9.5*
1527.2	6.2	5.8	3.7*	5.5*	5.6		
1553.3		1.4*					
1575.4	2.7*	2.1*	1.8*	2.8*	3.8*	5.5*	4.7*
1610.4	8.6	9.0	7.1	10.3	11.0	8.6	18.8
1621.0		2.7*					
1631.3	4.2	4.5	3.4*		5.3	4.7*	

a An asterisk (*) indicates an observed band with an intensity at the lower edge of the detection limit, reducing the accuracy of *A*.

b Average standard error for *A* by concentration: 0.7.

c Average standard error for *A* by concentration: 0.7 (10^–19^ cm/molecule).

d Average standard error for *A* by concentration: 2.4 (10^–19^ cm/molecule).

e Average standard error for *A* by concentration: 2.1.

f Average standard error for *A* by concentration: 6.3.

g Average standard error for *A* by concentration: 6.6.

h Average standard error for *A* by concentration: 6.6.

**Figure 7:**
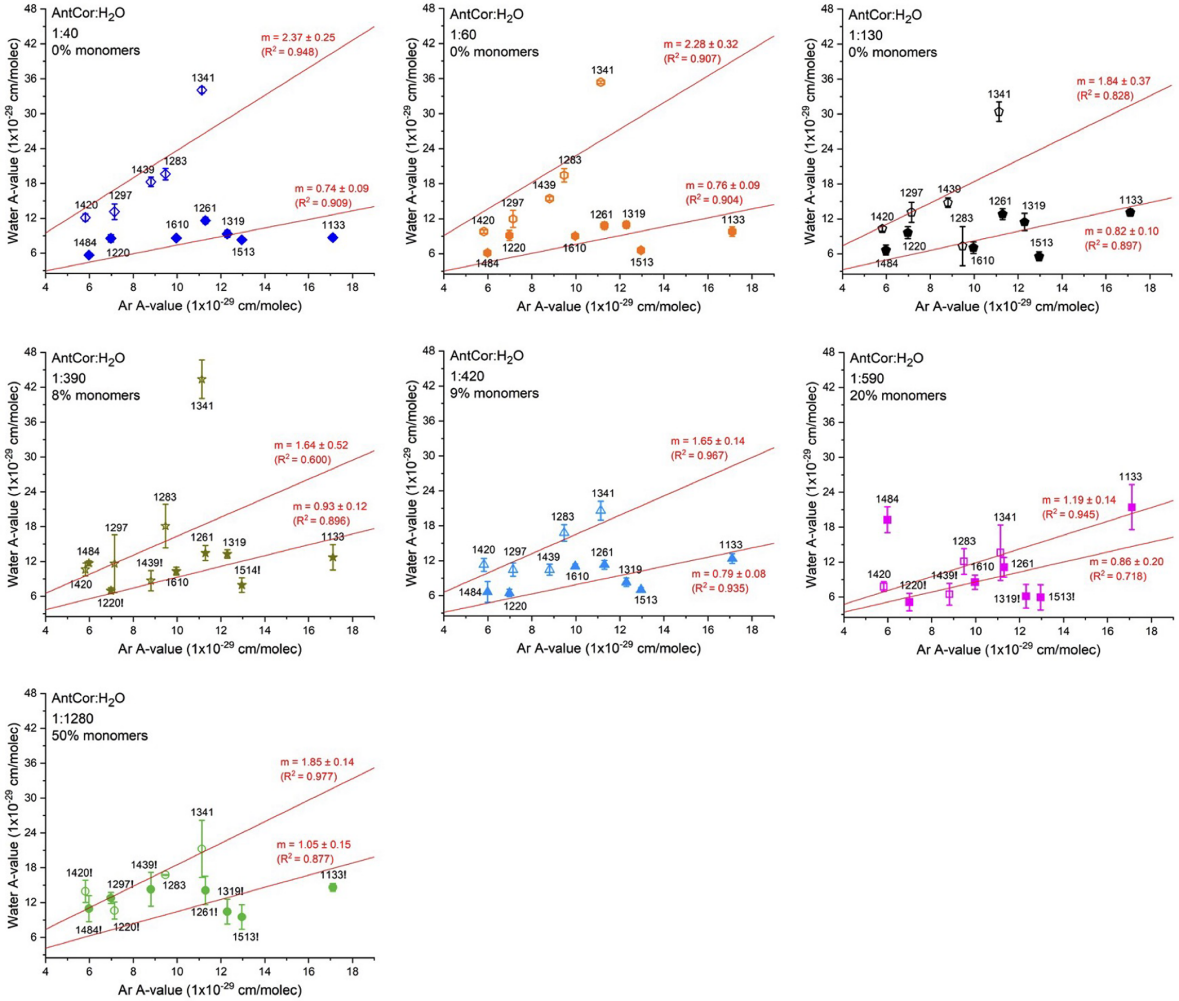
Comparison of *A* values of unirradiated AntCor bands in water and in Ar matrices for each AntCor:H_2_O concentration. The top fit line was determined using the bands at 1283, 1296, 1341, 1420, and 1439 cm^–1^ (open symbols), while the bottom fit line was determined using the 1134, 1220, 1261, 1319, 1484, 1515, and 1610 cm^–1^ bands (filled symbols). Error bars represent the standard error from the AntCor:H_2_O *A* calculation. Band positions followed by “!” are bands that were observed but were so small that their areas cannot be accurately determined.

A previous study^22^ found that the values of *A* for unirradiated Cor in water were approximately 85% of those for unirradiated Cor in an Ar matrix, across all Cor:H_2_O concentrations. To determine the ratio between *A* for AntCor in water and Ar, the values given in Table 2 were compared to those of unirradiated AntCor in Ar ice. Figure 7 shows the relationship using the unirradiated AntCor bands at 1133, 1220, 1261, 1283, 1297, 1319, 1341, 1420, 1439, 1484, 1513, and 1610 cm^–1^ across all concentrations. The ratio of *A* values fall into two groups. One group, composed of the 1283, 1296, 1420, and 1439 cm^–1^ bands, exhibit a linear fit with a large slope, while the second group, composed of the bands at 1133, 1220, 1261, 1319, 1484, 1513, and 1610 cm^–1^, exhibits a linear fit with a small slope. For the first group the slope of fit changes significantly with the PAH concentration, decreasing from 2.37 at 1:60 to 1.85 at 1:1280. In contrast, the second group has a smaller range in slopes, from 0.74 to 1.05. This indicates that the *A* values of the first group of bands, which can be attributed to the Ant portion of AntCor (Table 1), varies with concentration whereas the *A* values of the second group of bands, which can be attributed to the Cor portion (see Table 1), are not affected as much by concentration. While the slope of the first group of bands in Figure 7 does generally decrease as the PAH concentration decreases, there is a sudden change at the 1:590 concentration. The slope drops from 1.84 at 1:130 to between 1.64 and 1.19 for concentrations 1:390 through 1:590. After 1:590, there is a shift in “solvation” where an AntCor molecule is surrounded by more waters than by other AntCor molecules. At that point, the slope fit goes back up to 1.85 at AntCor:H_2_O 1:1280. As was discussed for Figure 5, there are more AntCor molecules present in the ice as monomers in starting from these concentrations, and thus there is a significant molecular rearrangement for increased water solvation.

The values for 1341 cm^–1^ appear to be well outside the range of the other bands for the highest concentrations (i.e., 1:40 to 1:390) in Figure 7. The 1341 cm^–1^ band involves concerted C–H in-plane bending modes on the Ant portion along with a duo of in-plane C–H bending modes on the Cor portion (see Figure 4). The large increase in the band’s intensity with increasing AntCor concentration might be due to a cooperative effect between AntCor molecules in a cluster, with the in-plane motions occurring in sync, creating a large change in dipole moment.

## 4 Photochemistry of AntCor in Water Ice

### 4.1 Effects of Concentration on AntCor Photoproducts

**Figure 8:**
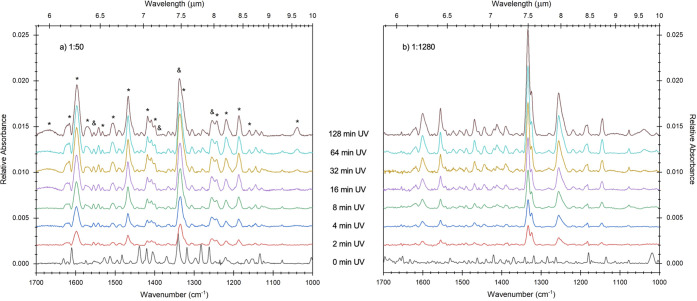
Mid-IR spectra from 1700 to 1000 cm^–1^ of AntCor:H_2_O at concentrations of (a) 1:60 and (b) 1:1280 throughout 128 min of UV irradiation. The asterisks (*) in (a) mark photoproduct bands that had a decrease in relative intensity between concentrations, while the bands marked with an ampersand (&) are those with an increase in relative intensity at lower PAH concentrations. Table 3 gives the bands and their relative intensities. The spectra shown are subtraction spectra (AntCor_UV_ – AntCor_unirrad_) as described in Section 2.1, where the unirradiated bands (0 min UV spectrum) have been removed. Spectra are baseline-corrected, filtered, and vertically offset for presentation.

The irradiation of AntCor:H_2_O ice was repeated for multiple concentrations, ranging from 1:40 to 1:1280, as mentioned earlier in Section 2.3. A comparison between the subtracted spectrum of the 128 min irradiated AntCor:H_2_O and the spectrum of the unirradiated sample is shown in Figure 8 for both the 1:60 and 1:1280 concentrations. The full MIR spectra for irradiated AntCor:H_2_O at each concentration are provided in Figures S1 and S2 in the Supporting Information. Concentrations with more AntCor clustering produce more photoproduct bands; the 1:60 (0% monomer fraction) concentration has a total of 37 bands, while the 1:1280 concentration (50% monomer fraction) has only 34 observed bands. The spectra for the two concentrations are considerably different, indicating that the PAH:H_2_O ratio significantly affects the chemistry. The bands at 1553, 1327, and 1255 cm^–1^, previously attributed to AntCor^+^,^43^ grow significantly for the 1:1280 concentration but only slightly for 1:60, consistent with a larger production of monomer cations at the low PAH concentration. For the case of 1:60, clusters of AntCor^+^ form and the amount of positive charges would increase with irradiation time. The positive charges would then induce a dipole moment in the surrounding neutral AntCor molecules, producing a frequency shift in their bands. The bands in high PAH concentration spectra collected after UV irradiation are shifted to lower wavenumbers in comparison to the spectrum of the unirradiated sample, and the intensities of the photoproduct bands grow steadily during the first 32 min of irradiation. Two new features appear after 16 min, located at 1040 and 1650 cm^–1^ (Figure 8). The band centered at 1650 cm^–1^ is very broad, which is consistent with an OH functional group. Secondary alcohols formed by the reaction of an OH radical with an AntCor unit are plausible candidates for these photoproduct bands. A previous study on the UV photolysis of a PAH in water ice layered with O_2_ attributed the 1040 cm^–1^ band to O_3_.^45^ Since our experiments do not have such oxygen-rich environments, it is more likely that the 1040 cm^–1^ band results from a photoproduct mode instead of O_3_.

### 4.2 Identification of AntCor Photoproducts

**Figure 9:**
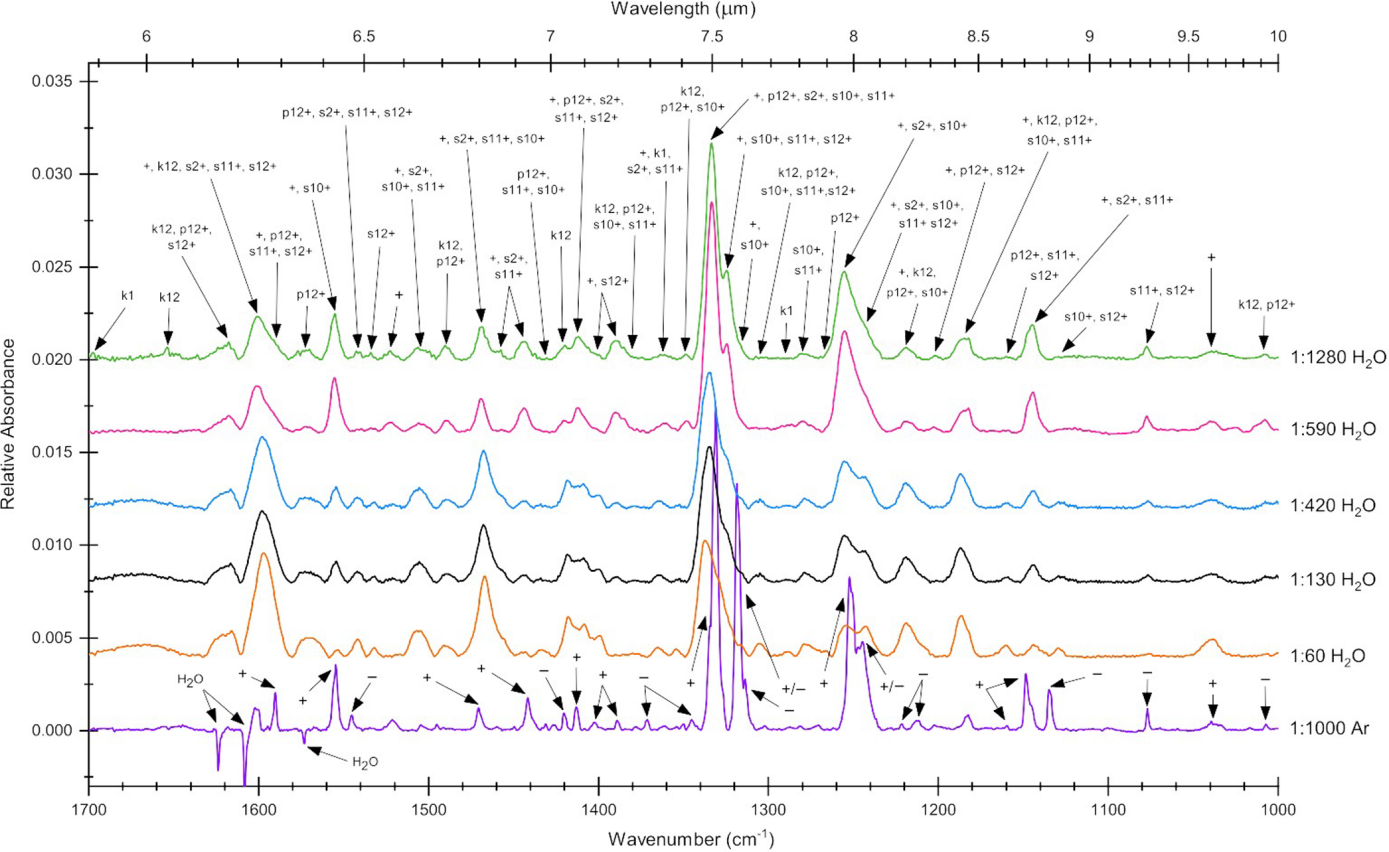
Difference spectra between 1700 and 1000 cm^–1^ of AntCor:H_2_O at concentrations of 1:60 (orange), 1:130 (black), 1:420 (blue), 1:590 (pink), and 1:1280 (green) and of AntCor:Ar (purple). AntCor:H_2_O photoproduct peaks have been labeled with arrows according to their possible photoproduct species (Figure 3), while the AntCor cation (+) and anion (−) peaks identified in prior work^43^ are labeled on the Ar matrix spectrum. ± bands are superimposed cation and anion bands. All AntCor:H_2_O spectra were collected after 128 min of UV irradiation, while the AntCor:Ar spectrum was collected after 32 min of UV. The AntCor:Ar spectrum has been reduced by a factor of 3 for display on the same scale as the smaller peaks in water. Spectra have had unirradiated species subtracted off and been baseline-corrected, filtered, and artificially offset for presentation.

In Table 3 we report the photoproduct band positions, the relative intensities, the theoretical band positions and intensities, and the assignments for the 1:60 and 1:1280 concentrations after 128 min of UV irradiation. Difference spectra are given in Figure 9. Using the cation assignments from a previous study^43^ as a starting point, the subtraction spectra of the irradiated samples were compared to the theoretically calculated band positions and relative intensities (described in Section 2.3) for AntCor^+^. Given that most of the photochemistry occurs at low PAH concentrations, when there are more monomers, we use the 1:1280 concentration to identify the photoproducts. The irradiated 1:1280 spectrum closely matches the theoretical spectrum of AntCor^+^ in both band positions and relative intensities. The AntCor^+^ bands from irradiated AntCor:H_2_O are at 1039, 1144, 1186, 1202, 1219, 1243, 1255, 1316, 1327, 1336, 1363, 1389, 1399, 1408, 1444, 1456, 1467, 1505, 1524, 1554, 1589, and 1597 cm^–1^. Many of the calculated AntCor^+^ bands have already been confirmed experimentally,^46^ although some were mixed with anion bands. Anion species are unlikely to form in water ice, as electrons are trapped and stabilized in water ices. This explains the dramatic difference in relative band intensities between the water and Ar spectra. Almost all of the AntCor^+^ bands overlap to some extent with bands from other oxygenated and protonated photoproducts.

**Table 3:** AntCor:H_2_O Photoproduct Bands (±2 cm^–1^) between 1700 and 1000 cm^–1^ with Relative Intensity Values and Assignments Determined via Comparison to Theoretical Spectra and Previous Studies on AntCor:Ar (a), Cor:H_2_O,^22,43^ and Ant:H_2_O^21^ after 128 min of UV Irradiation^a^

	rel intens				
position in H_2_O (cm^–1^)	1:60	1:1280	theor predicted bands (cm^–1^)	intensity of theor bands (10^–18^ cm/molecule)	assignment
1006.4	0.06	0.03	1007.7, 1015.3	8.4, 15.5	k12, p12+
1039.1	0.17	0.08	1040.9(a)	4.5(a)	+
1076.6	0.03	0.03	1076.0, 1072.0	7.5, 4.9	s11+, s12+
1129.0	0.05		1133.8, 1126.1	2.9, 5.7	s10+, s12+
1144.2	0.07	0.12	1147.9(a), 1149.4, 1151.2	38.1(a), 103.6, 169.0	+, s2+, s11+
1160.5	0.10		1154.5, 1158.6, 1161.5	7.5, 30.8, 75.4	p12+, s11+, s12+
1186.4	0.37	0.10	1182.3(a), 1188.4, 1184.1, 1183.0, 1183.9	18.3(a), 13.8, 29.6, 41.5, 30.6	+, k12, p12+, s10+, s11+
1202.8(sh)	0.05	0.02	1211.6, 1204.9, 1211.3	0.7, 31.3, 28.4	+, p12+, s12+
1219.2	0.27	0.06	1219.3, 1217.8, 1220.7, 1218.9	9.1, 31.9, 23.3, 19.4	+, k12, p12+, s10+
1243.0(sh)	0.24	0.15	1245.2(a), 1241.5, 1241.5, 1245.3, 1239.3, 1241.3	106.1, 32.2, 26.6, 67.4, 124.8	+, s2+, s10+, s11+, s12+
1255.0	0.26	0.45	1252.3(a), 1252.0, 1252.2	193.3(a), 108.7, 81.1	+, s2+, s10+
1265.6	0.03		1269.0	8.0	p12+
1279.7	0.10	0.03	1270.2, 1283.4	3.3, 9.9	s10+, s11+
1288.7	0.04		1285.5	13.0	k1
1306.8	0.10	0.03	1305.7, 1304.2, 1296.5, 1304.3, 1306.2	12.3, 52.3, 17.2, 85.5, 5.9	k12, p12+, s10+, s11+, s12+
1316.1(sh)	0.05		1318.8(a), 1316.0	35.0(a), 108.1	+, s10+
1327.0(sh)	0.47	0.35	1330.9(a), 1330.6, 1329.9, 1330.5	211.0(a), 124.3, 223.6, 199.1	+, s10+, s11+, s12+
1336.3	1.00	1.00	1334.5, 1337.2, 1338.7, 1338.7, 1341.2, 1339.1	1.0, 13.2, 25.5, 9.5, 7.0	+, p12+, s2+, s10+, s11+
1353.0	0.04	0.07	1356.5, 1357.5, 1349.7	7.7, 37.3, 16.0	k12, p12+, s10+
1363.9	0.06	0.04	1367.2, 1367.3, 1368.9, 1369.7	23.0, 10.9, 102.3, 67.0	+, k1, s2+, s11+
1377.5	0.04	0.02	1383.1, 1375.3, 1378.1, 1376.5	5.9, 15.6, 24.5, 23.5	k12, p12+, s10+, s11+
1389.7		0.09	1388.8(a), 1389.3	26.5(a), 32.5	+, s12+
1399.3(sh)	0.12	0.02	1402.1(a), 1397.0	12.9(a), 89.1	+, s12+
1408.3	0.20	0.13	1413.1(a), 1411.5, 1406.3, 1402.2, 1410.4	12.8(a), 10.6, 9.6, 20.7, 9.2	+, p12+, s2+, s11+, s12+
1417.7	0.31	0.05	1422.9	18.4	k12
1431.8	0.04		1434.2, 1436.9, 1432.8	17.9, 33.8, 16.5	p12+, s10+, s11+
1444.2	0.03	0.06	1441.1(a), 1443.8, 1448.8	33.6(a), 27.6, 10.7	+, s2+, s11+
1456.1(sh)	0.06	0.02	1454.8, 1458.3, 1458.6	2.3, 30.9, 10.7	+, s2+, s11+
1467.2	0.69	0.14	1470.9(a), 1474.7, 1472.8, 1469.6	20.8(a), 47.1, 27.2, 42.6	+, s2+, s10+, s11+
1490.2	0.09	0.05	1481.1, 1484.4	19.8, 59.0	k12, p12+
1505.5	0.24	0.06	1500.2, 1501.5, 1499.7, 1500.8	22.9, 57.7, 47.4, 23.1	+, s2+, s10+, s11+
1524.7	0.03	0.05	1523.1	24.8	+
1532.4	0.11	0.01	1535.7	44.9	s12+
1541.3(sh)	0.01	0.02	1539.5, 1542.2, 1542.2, 1544.9	133.4, 46.9, 202.0, 139.3	p12+, s2+, s11+, s12+
1554.1	0.04	0.16	1554.9(a), 1551.5	94.5(a), 100.5	+, s10+
1570.1	0.19	0.06	1565.6	8.1	p12+
1589.3(sh)		0.06	1589.8(a), 1587.2, 1592.0, 1591.4	12.8(a), 119.6, 17.7, 49.4	+, p12+, s11+, s12+
1597.5	1.27	0.34	1601.6(a), 1591.1, 1601.0, 1666.8, 1591.4	40.5(a), 70.8, 99.8, 44.4, 49.4	+, k12, s2+, s11+, s12+
1615.2	0.64	0.11	1613.4, 1613.2, 1615.1	28.9, 22.6, 44.9	k12, p12+, s12+
1662.2	0.33	0.02	1656.4	136.3	k12
1697.0		0.01	1702.9	60.0	k1

a The structures of the photoproducts are shown in Figure 3. Bands that appear as shoulders on neighboring bands are indicated by “(sh)”.

In addition to AntCor^+^, we identified the photoproducts shown in Figure 3. They include positively charged aromatic alcohols (s2+, s10+, s11+, s12+), protonated AntCor (p12+), and ketones (k1, k12). These photoproducts form after the addition of UV photons (*h*ν) to H_2_O ice generates H and OH radicals (eq 3) and ionizes AntCor in a one-photon process (eq 4). AntCor^+^ can then recombine with an electron to re-form AntCor or react with an OH or H radical to form cationic alcohols (eq 5) or protonated AntCor (eq 6), respectively. These reactions are barrierles,s as they involve a radical–radical mechanism. The relative energies for the cationic alcohols, in order of least to most stable conformation, are as follows: 0.0 kcal/mol for s12+, 0.18 kcal/mol for s10+, 2.22 kcal/mol for s2+, and 3.21 kcal/mol for s11+. It should be noted that these energies are from gas-phase calculations, where the OH groups exclusively form *intra*molecular hydrogen bonds. In water ice, the alcohols could form *inter*molecular hydrogen bonds as well. The formation of ketones occurs from the addition of an H radical to a PAH alcohol. Reactions with neutral AntCor molecules, such as H addition, can be expected to have non-negligible energy barriers.^47^ Our results are consistent with previous experiments,^21,22,48^ as the formation of cations, alcohols, protonated PAHs, and ketones occurs for other PAHs as well.
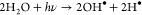
3
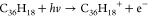
4
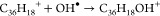
5
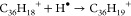
6

Our assignments were made by comparison with computed spectra, and photoproduct identifications were made when all the computed bands for a given molecule matched the experimental bands. Cationic aromatic alcohols form as their ionization potentials are comparable to that of AntCor (∼6.23 eV). They have bands with strong intensities in the 1140–1255, 1310–1400, and 1530–1560 cm^–1^ ranges that overlap with the bands of AntCor^+^. Only a few bands can be attributed solely to alcohols: 1076, 1129, 1279, and 1532 cm^–1^. Protonated AntCor (p12+) has strong bands in the 1530–1615 cm^–1^ region, and the band at 1565 cm^–1^ can be attributed purely to p12+. Neutral ketones have very strong bands in the 1600–1700 cm^–1^ range. On the basis of the intensity of the AntCor photoproduct bands in the 1600–1700 cm^–1^ region, ketones are formed in very small amounts. We did not detect diols or quinones, possibly because of the higher PAH concentrations involved in our experiments in comparison to prior studies. We also did not detect any photodimers formed by UV photodimerization of two AntCor molecules.

A band appeared in the data at around 2234 cm^–1^ (see Figures S1 and S2 in the Supporting Information) that did not seem to be a contamination band or a byproduct of PAH:H_2_O photolysis. Likewise, this signal did not lie in the AntCor:H_2_O photoproduct range (1700–1000 cm^–1^) and had not appeared in AntCor:Ar spectra previously.^43^ Other works have reported a band at 2233 cm^–1^ from carbon suboxide (C_3_O_2_) when AntCor:H_2_O was in water ice at 18 K,^46,49^ only 1.5 cm^–1^ away from the band we observed in AntCor:H_2_O. This C_3_O_2_ feature is due to the ν_3_ CCO asymmetric stretching mode.^46^ The ν_3_ band characteristic of carbon suboxide can be found within the 2500–2000 cm^–1^ range, with variation in the specific position dependent on the concentration of C_3_O_2_ in the ice matrix. C_3_O_2_ was likely produced from the irradiated AntCor:H_2_O sample as a product of CO_2_, as there was no C_3_O_2_ band prior to irradiation.

### 4.3 Near-Infrared Region

**Figure 10:**
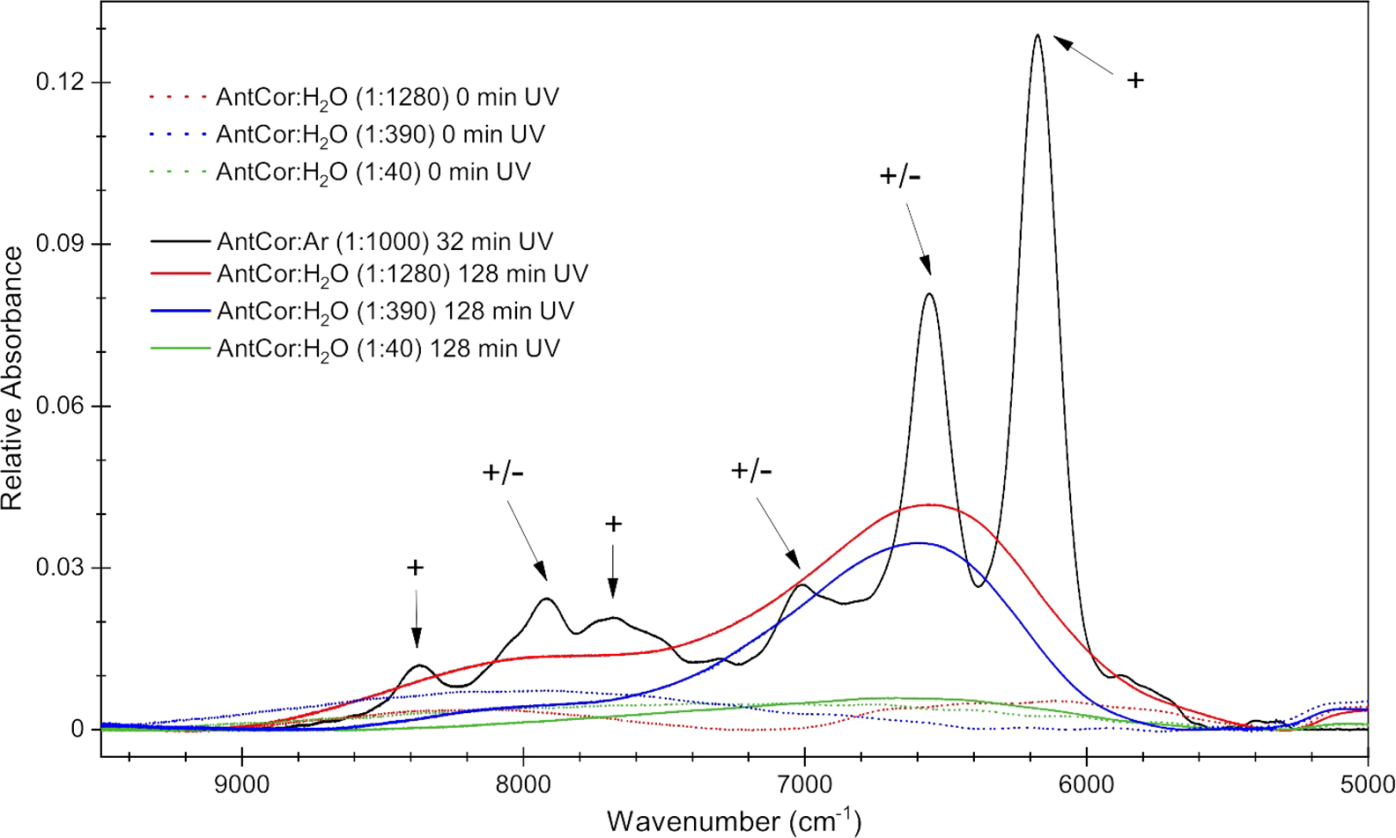
NIR spectra (from 9500 to 5000 cm^–1^) before irradiation of AntCor samples (dotted lines) and after UV irradiation (solid lines) in both water and Ar ice, at different concentrations. The spectrum of unirradiated AntCor:Ar is not available. However, AntCor cation and anion peaks^43^ are indicated on the photolyzed AntCor:Ar spectrum. All spectra have been baseline-corrected for presentation within the same absorbance range.

The bands in the near-infrared wavelength regions are due to electronic transitions of AntCor^+^. Therefore, these bands provide evidence of ion-mediated chemistry. The near-infrared (NIR) spectra of the UV-irradiated sample in both water and Ar are compared in Figure 10. When AntCor is irradiated in an Ar matrix,^43^ strong peaks appear in the 9000–6000 cm^–1^ range, with particularly intense and well-defined bands at approximately 6200 and 6600 cm^–1^ and less intense bands clustered between 8500 and 7250 cm^–1^. By extending the AntCor:H_2_O sample scans to the NIR region, it can be seen that bands do appear within the same region as in the Ar matrix. Water broadens bands, and therefore the AntCor:H_2_O photoproduct bands can only be resolved as two bands, whereas the Ar data exhibit around nine bands. However, the bands in water follow a relative intensity trend similar to those in Ar, with the most intense band being between 7000 and 6000 cm^–1^ and a smaller band being centered around 8000 cm^–1^.

Figure 10 shows the relationship between the AntCor^+^ band intensity in the NIR and AntCor:H_2_O concentrations. The spectra of the unirradiated AntCor:H_2_O samples only show undulations in the baseline, as neutral PAHs do not produce absorption features in this region.^50^ At 1:1280, two broad bands are clearly seen in the spectrum of the irradiated sample. However, once a high AntCor concentration, such as 1:40, is reached, only one very broad band of low intensity is observed. The width of this NIR band encompasses the positions of the two peaks seen at lower AntCor concentrations, and it could be that at such low intensity the two bands merged to become indistinguishable. The band at around 6600 cm^–1^ has the greatest intensity in the 1:1280 concentration. In the 1:390 concentration this band’s absorbance is half that of the 1:1280 value, and in the 1:40 concentration the absorbance is only a fifth of the 1:1280 value. The low intensity of cation bands in the spectra of the 1:40 concentration reveals how challenging AntCor^+^ formation is at high PAH concentrations, supporting what was discussed in Section 4.2 regarding the PAH:H_2_O concentration effects in the MIR region. That is, at high PAH concentrations there are not enough water molecules to stabilize the electrons that are produced during PAH cation formation, and therefore the electrons will simply recombine with the PAH ions. The increase in the intensity of NIR bands with lower PAH concentrations in water ice agrees with prior work,^1^ which indicated that the ionization efficiency increases as the concentration decreases.

As was previously discussed for the MIR region (Section 4.1), the change in a band’s absorbance growth between concentrations can tell us about the photoproduct species, as well as clustering or isolation of the PAH molecule. The difference in the amount of change between concentrations suggests that the production of the 6600 cm^–1^ band has an optimal concentration where it can reach its maximum absorbance. This could, for example, be the consequence of the ionization efficiency and isolation of AntCor reaching an optimal concentration, where most of the sample becomes ionized immediately, resulting in a larger 6600 cm^–1^ peak. Given the values shown in Figure 10, the concentration that produces the most intense 6600 cm^–1^ band is likely 1:1280 or even more dilute. AntCor^+^ was previously reported^43^ to have the lowest-energy electronic transition of any large PAH with its band at 6175 cm^–1^, the dibenzopolyacene family being an exception.^51^ AntCor:H_2_O also contains this strong feature, though the band has been broadened and shifted to higher wavenumbers, between 6800 and 6500 cm^–1^. This is due to a change in the electrostatic and structural properties between the water ice and argon matrices. We were unable to find any previous studies of PAHs in water that reported bands in the NIR range, and therefore AntCor^+^ appears to have the lowest energy electronic transition reported of PAHs in water ice.

## 5 Astrophysical Implications

### 5.1 Photoproduct Production

Most current protoplanetary disk models treat IDPs as homogeneous surfaces, when in reality, a grain’s mantle and surface have different chemistries. Previous studies have discussed how the ice mantle surrounding a dust grain experiences adsorption and desorption yet the interface between the mantle and the dust’s core material can have different desorption and diffusion properties.^52^ This work provides insights into the contribution of PAHs to the protoplanetary disk mantle chemistry. The rate constant, *k*, for the production of each AntCor:H_2_O photoproduct was determined from the growth of the integrated band area as a function of UV photolysis time using the 1:1280 ratio. The rate constant *k*_AntCor^+^_ = (3.450 ± 1.568) × 10^–4^ s^–1^ was determined from the 1524 cm^–1^ band growth fit with an exponential curve as a first-order reaction. The other rate constants are *k*_alcohol^+^_ = (6.926 ± 2.418) × 10^–4^ s^–1^ (1077 cm^–1^, first order), *k*_H^+^_ = (4.325 ± 1.145) × 10^–4^ s^–1^ (1570 cm^–1^, first order), and *k*_CO_2__ = (3.623 ± 0.124) × 10^–6^ M s^–1^ (2341 cm^–1^, zeroeth order). Prior works on photolysis of PAHs in water ice have assumed first-order reactions,^53,54^ which we see here for AntCor. The production of a pyrene cation, Py^+^, in water ice was reported as *k* ≈ 10^–3^ s^–1^ for concentrations between 1:5000 and 1:10000. That rate is 1 order of magnitude greater than what we see for AntCor^+^ (1:1280). A study on coronene showed that for a PAH:H_2_O ratio of 1:14000 the ionization yield is 60% and for a ratio of 1:1100 the yield drops to 12%.^55^ For AntCor^+^, the ionization yield could be even smaller than 12%, given its smaller monomer fraction in comparison with Cor. This could explain the difference in the magnitudes of the rate constants.

Carbon dioxide emissions have been observed in protoplanetary disks and high-mass protostars. The CO_2_ abundances in these environments are much less than what is seen in the ISM; however, an analysis^52^ indicates that the amount of CO_2_ in ice on grains increases as the distance from the disk center decreases. With strong modes, CO_2_ presents a unique identifier with which to trace disk evolution. CO_2_ is mainly formed in ice by grain-mediated reactions in regions where FUV photons (*h*ν) photodissociate H_2_O-coated dust grains (eq 3) and the products then react with the carbons on the PAH to form carbon dioxide (eq 7). After that, photo- and chemical desorption of CO_2_ enhances its abundance in the gas phase. The amount of CO_2_ in the gas phase peaks at *r* ≤ 10 AU in the disk midplane, where the temperature is high enough to overcome the endothermicity of the reaction.
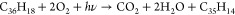
7

**Figure 11:**
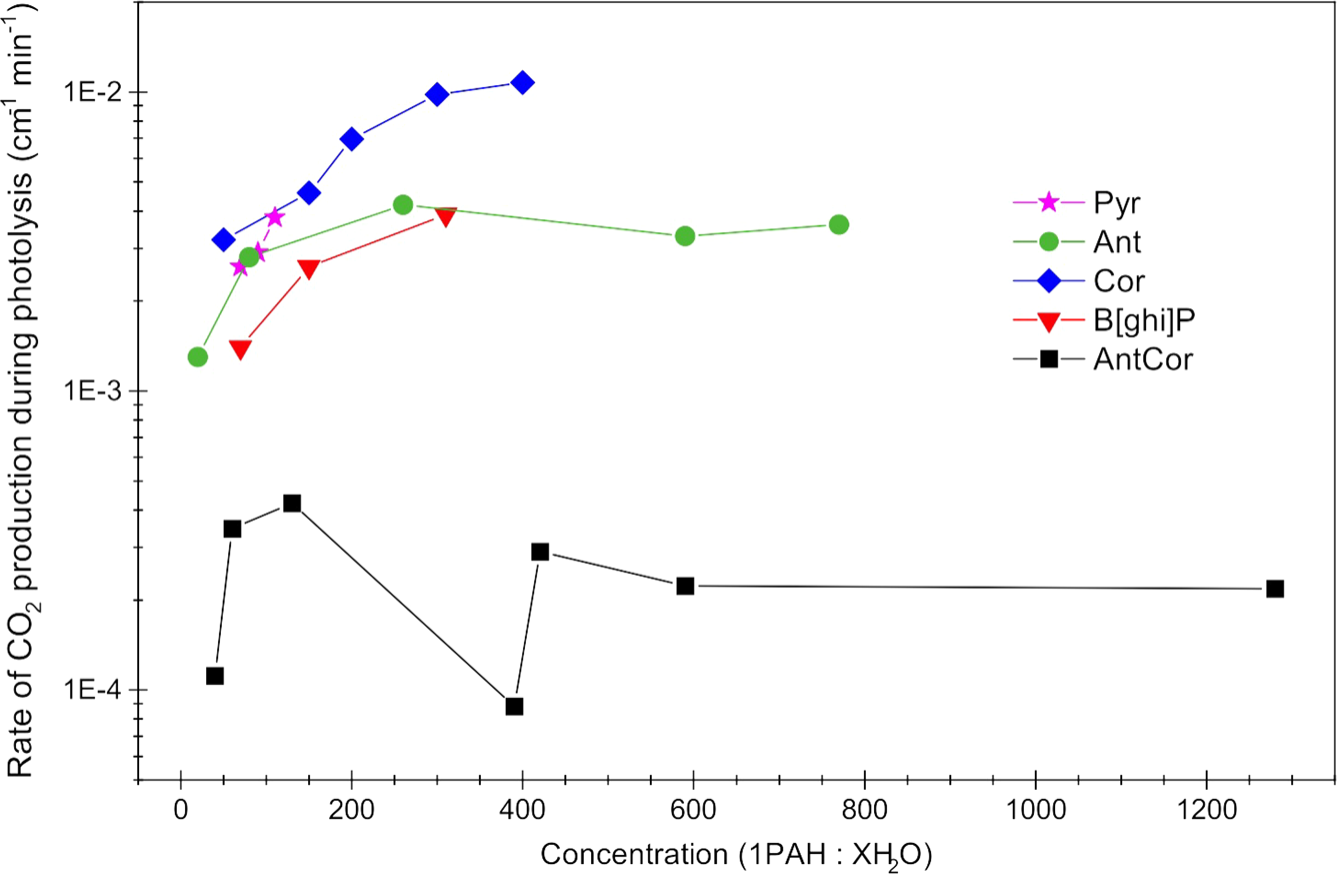
Growth of the 2341 cm^–1^ CO_2_ band area, in experiments with different PAHs, as a function of UV photolysis time plotted against the PAH:H_2_O concentration. Pyrene (Pyr) and benzo[*ghi*]perylene (B_*ghi*_P) experiments did not include low PAH concentrations.^21^

The degree of PAH degradation into CO_2_ has been quantified before^21,22^ via plots of unirradiated PAH band decay with irradiation alongside growth in the integrated areas for CO_2_ modes. Due to the large number of photoproduct bands produced by AntCor in water, quantifying the degradation of the unirradiated sample proved difficult, as there was significant overlap between the bands of the unirradiated and irradiated samples. Instead, we used the growth of the CO_2_ band at 2342 cm^–1^ as a proxy for the degradation of unirradiated AntCor molecules. To do this, the integrated area of the CO_2_ band throughout irradiation was normalized to its band area after 2 min of UV irradiation.^21^ This eliminates any interference from residual CO_2_ in the chamber at the start of the experiment. As shown in Figure 11, AntCor does not follow the CO_2_ production rate observed for other PAH:H_2_O ice experiments. Prior PAH:H_2_O experiments indicated that the production of CO_2_ is linear with respect to PAH:H_2_O concentration, with slopes on the order of 10^–3^–10^–2^ cm^–1^ min^–1^ (Table 4) for concentrations of less than 1:600.^21^ While CO_2_ production for a given concentration of AntCor is linear, the slopes for the integrated absorbance are 1 order of magnitude lower than those observed for smaller PAHs (i.e., 10^–4^). These results are also consistent with a lower production of AntCor^+^, cationic alcohols, and protonated AntCor. As was discussed earlier, the monomer fractions for Cor and Ant are much larger than for AntCor, meaning that Cor and Ant are surrounded by more water molecules than is AntCor, and consequentially their production of CO_2_ is considerably larger.

**Table 4:** Slopes (cm^–1^ min^–1^) of the 2341 cm^–1^ CO_2_ Band Area Growth during UV Photolysis for Different PAHs and PAH:H_2_O Concentrations

Ant:H_2_O	slope^21^	Py:H_2_O	slope^21^	Cor:H_2_O	slope^22^	B_*ghi*_P:H_2_O	slope^21^	AntCor:H_2_O	slope
1:20	0.0013							1:40	0.000114
1:80	0.0028	1:70	0.0026	1:50	0.00321	1:70	0.0014	1:60	0.000339
		1:90	0.0029						
		1:110	0.0038	1:150	0.0046	1:150	0.0026	1:130	0.000420
1:260	0.0042			1:200	0.00695				
				1:300	0.0098	1:310	0.0039	1:390	0.000104
				1:400	0.01078			1:420	0.000257
1:590	0.0042							1:590	0.000260
1:770	0.0036							1:1280	0.000217

As shown in Figure 11, CO_2_ production from a PAH in water ice generally tends to increase with decreasing PAH concentration. Only two of the PAHs studied thus far, Ant and AntCor, investigated CO_2_ production at low PAH concentrations. In both cases, the CO_2_ production rate decreases as the concentration passes the 1:400 mark. The production of CO_2_ in AntCor:H_2_O increases until it reaches the 1:390 concentration, at which point there is a sharp decrease that coincides with an inflection point in the photochemistry as it goes from molecular to ion mediated.^52^ In the high-PAH-concentration regime, AntCor molecules are clustered together with limited exposure to water molecules; therefore, the CO_2_ production that occurs happens at the cluster edges. The dramatic change in the CO_2_ production rate at 1:390 is due to the start of the low-PAH-concentration regime, where there are more AntCor monomers and thus there is greater opportunity for water molecules to access and react with AntCor. While this data point might seem like a discontinuity, it demonstrates the transition of AntCor:H_2_O chemistry on moving from the high (concentrations 1:40 through 1:130) to low (1:390 through 1:1280) PAH regimes. As this new low PAH regime starts, the CO_2_ production again increases and then plateaus. The difference between the two regimes is magnified for AntCor due to the larger and irregular PAH size.

### 5.2 Components of Astronomical Spectra

**Figure 12:**
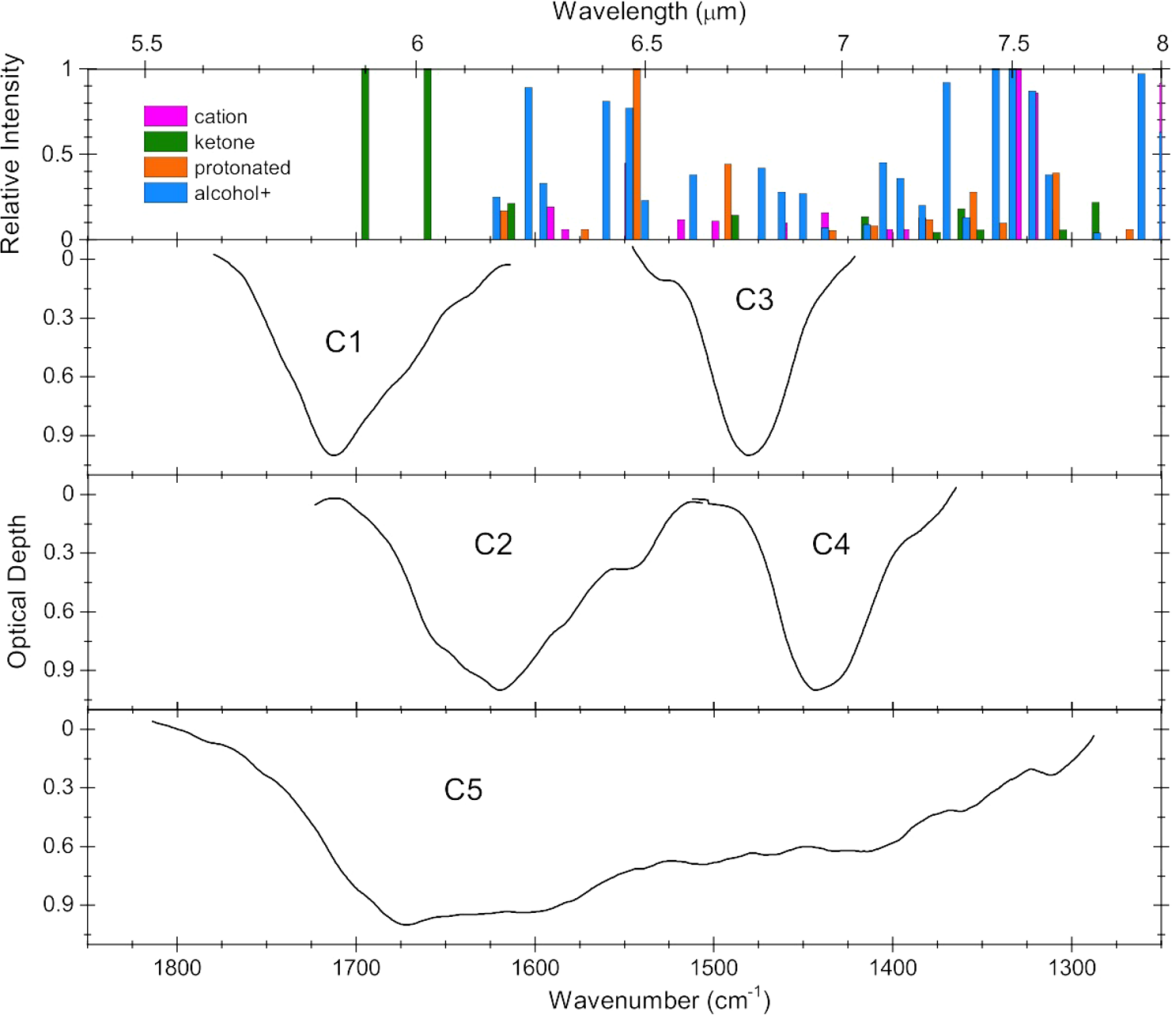
Comparison between AntCor:H_2_O photoproduct band positions and astronomical spectra. (top panel) AntCor:H_2_O photoproducts as given in Table 3, with species denoted by color. The relative intensity of a band within the species’ spectrum is indicated by the bar height. (lower panels) The C1–C5 residual components identified in the spectra of embedded YSOs without the 6.0 μm H_2_O feature.^2^

Figure 12 shows that photoproducts from VUV irradiation of AntCor in water ice can contribute to the C1–C5 astronomical absorption features identified by Boogert present in YSOs^2^ (bottom three panels). Using the photoproduct band assignments from Table 3, the AntCor:H_2_O photoproduct bands were lined up with the C1–C5 residual components while the cation bands aligned with the C5 residual component. The PAH cation features in earlier studies^21,22^ cluster within the C2 region and the C4 region for Cor, while AntCor^+^ bands are more evenly distributed across the 6–7.6 μm region that encompasses C2, C3, C4, and C5. Figure 12 shows that the C5 component has a non-negligible intensity at around 7.5 μm, while Figure 8b demonstrates that the strongest AntCor photoproduct bands are between 7.4 and 8.0 μm and are influenced by AntCor:H_2_O concentration. AntCor^+^ is the predominant photoproduct at low PAH concentrations, with the most intense AntCor^+^ bands falling at the lower edge of C5 (<1350 cm^–1^, <7.3 μm). Other photoproduct species such as ketones and protonated AntCor contribute less to the bands in the 7.5–8.0 μm region, but have more intensity in comparison to the cation bands in the 5.8–7.5 μm region. Photoproduct bands from ketones are most intense within the C1 region, with some weaker bands spread throughout the spectrum. Positively charged AntCor alcohols are rather evenly spread out within the 6.1–8.0 μm range (∼1650–1250 cm^–1^), matching the C5 component best. Protonated AntCor bands are also spread out, with the most intense bands of the species lining up with C2 and some weaker bands grouping within the C5 range. One should be able to use the intensity between 7.5 and 8.0 μm from observations to constrain the degree to which all the photoproducts contribute to the astronomical absorption between 6.0 and 8.0 μm via relative intensities.

## 6 Conclusions

This study reports the first analysis of AntCor spectra and its UV-induced chemistry in water ice. An initial study of AntCor analyzed AntCor in an inert argon matrix, thereby mimicking gas-phase conditions in the interstellar medium.^43^ The data from this study allow AntCor results to be applied to dense cloud environments in astronomical research. This work contributes to the variety of unirradiated and ionized PAH spectra available from the Experimental Library of the NASA Ames PAH IR Spectral Database (PAHdb)^39,56,57^ studied using the matrix isolation method. Future directions for AntCor study are its suspension in crystalline water ice to simulate the Solar System icy bodies, with special attention to PAH clustering,^58^ and doping the water ice with other common molecules such as methane, nitrogen, CO, or CO_2_ for relevance to Pluto or other icy bodies in space.

In this study we found that the concentration of AntCor in H_2_O ices affects the nature and amount of the photoproducts formed. Unlike what was observed in prior studies, the *A* values of bands from unirradiated AntCor samples are split into two groups, and changes in concentration affect the two groups differently. The AntCor:H_2_O concentrations between 1:40 and 1:130 have the most clustering. For the unirradiated AntCor, these spectra are consistent with theoretical spectra of AntCor clusters. At the 1:420 concentration the spectral profile of unirradiated AntCor changes due to more water solvation and the structural reorganization of AntCor clusters.

For UV-irradiated AntCor:H_2_O, the effect of concentration is even more pronounced. At high PAH concentrations (e.g., 1:60) the spectrum shows that AntCor molecules are mostly clustered and increasingly ionized up to 32 min of UV irradiation. The production of secondary alcohols occurs after 16 min of UV irradiation. At low PAH concentrations (e.g., 1:1280) the scenario is quite different, with mostly AntCor^+^ production, some production of cationic aromatic alcohols, and a very small amount of protonated AntCor. The NIR spectra confirm that the production of AntCor^+^ increases with a decrease in PAH concentration. Ketones are also present after photolysis, but in a small amount. Unlike the case with Cor and Ant,^21,22^ dioxygenated species of AntCor do not form, as fewer water molecules can interact with the larger PAH, for a given concentration. From our data we were able to estimate rate constants for the first-order productions of AntCor^+^, cationic alcohols, ketones, and protonated AntCor that are on the magnitude of 10^–4^ s^–1^ for low PAH concentrations (1:1280). CO_2_ production from AntCor:H_2_O (1:1280) irradiation has a zeroth-order rate constant at around 10^–6^ absorbance/s.

The CO_2_ production from AntCor is much less than what was reported for Ant and Cor in previous studies, also due to the limited amount of water molecules that are alongside the PAH. We did observe a large decrease in CO_2_ production for the 1:390 concentration that corresponds to the change in chemical regime on going from high to low PAH concentration. As the PAH concentration continues to decrease further, moving toward 1:1280, more AntCor molecules become available to react, which leads to another increase in CO_2_ production. These results reinforce the need to consider different ice concentrations in the exploration of PAH contributions to astronomical models regarding CO_2_. UV irradiation of AntCor:H_2_O produces strong photoproduct bands between 7.4 and 8.0 μm (1350–1250 cm^–1^). When assessing how much influence AntCor makes in an observed astronomical spectrum, one can estimate the total amount of photoproducts via comparison with the observations in the 7.4–8.0 μm range.

## Supporting Information Available

Full MIR spectra for unirradiated and irradiated AntCor:H_2_O samples, at all concentrations (PDF)
