# Supplementary material for: Infrared Spectroscopy and Photochemistry of Anthracoronene in Cosmic Water Ice
Source: ACS Earth Space Chem. 2022 Jan 9;6(1):165–80. doi: 10.1021/acsearthspacechem.1c00337 (PMC8785219; doi:10.1021/acsearthspacechem.1c00337)
Supplement: Supplementary file 1 — sp1c00337_si_001.pdf [file sp1c00337_si_001.pdf]

Infrared Spectroscopy and Photochemistry of Anthracoronene in Cosmic Water-Ice

Julie M. Korsmeyer,[*,§,¶] Alessandra Ricca,[§,#] Gustavo A. Cruz-Diaz,[§,∆] Joseph E. Roser,[§,#] and Andrew L. Mattioda[§]

§NASA Ames Research Center, Mail Stop 245-6, Moffett Field, CA 94035-1000 USA

¶Department of Chemistry, University of Chicago, 5735 S. Ellis Ave., Chicago, IL 60627 USA

#Carl Sagan Center, SETI Institute, 399 Bernardo Ave., Suite 200, Mountain View, CA 94043 USA

∆BAER Institute, P.O. Box 25, Moffett Field, CA 94035-1000 USA

*E-mail: jkorsmeyer@uchicago.edu

Supporting Information:

The full MIR spectra for unirradiated and irradiated AntCor:H_2O samples, at all concentrations.

Figure S1 on page S2
Figure S2 on page S3

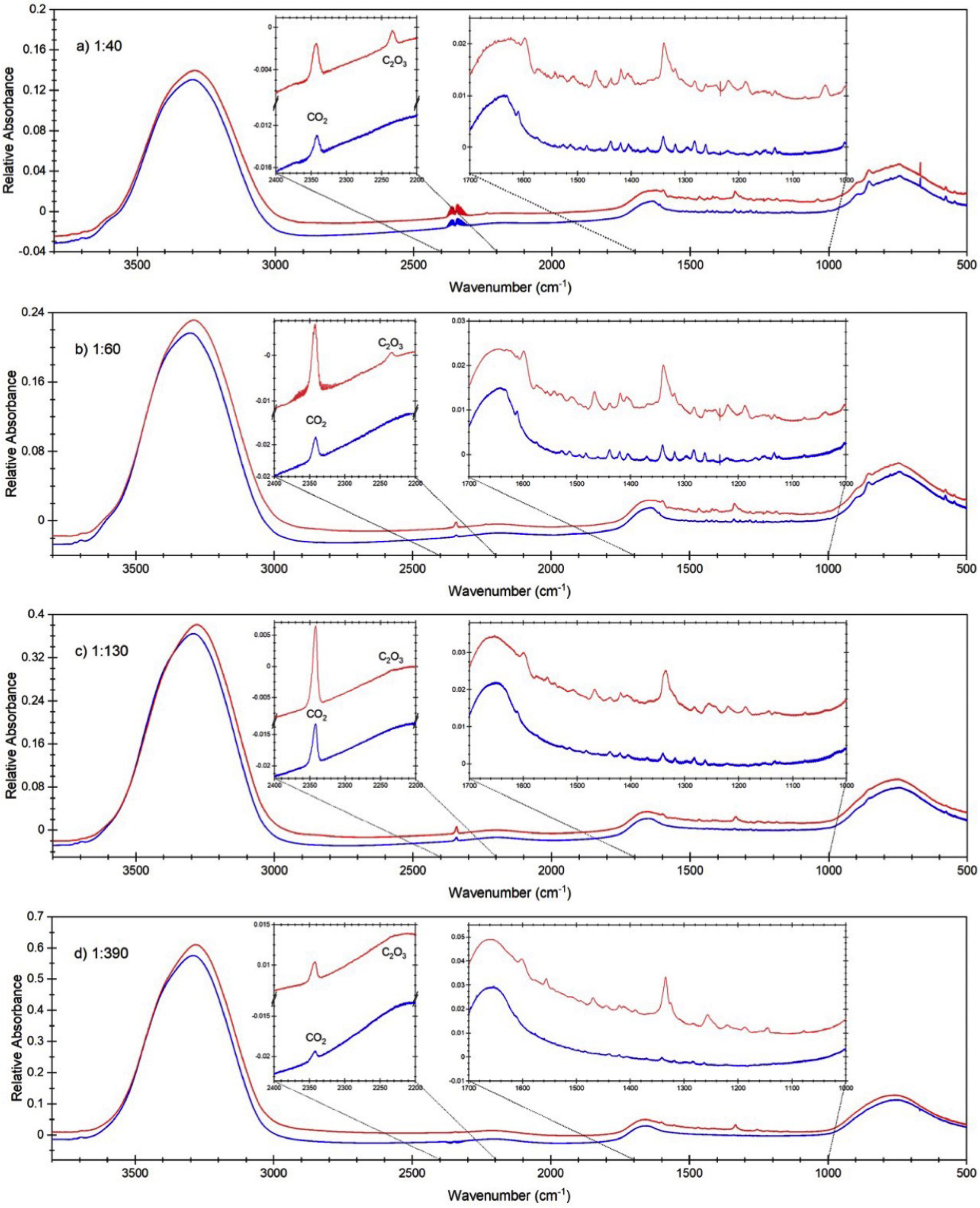

Figure S1: The full MIR spectra for unaltered unirradiated (blue) and irradiated (red) AntCor:H_2O samples, at concentrations a) 1:40, b) 1:60, c) 1:130, d) 1:390. The spectra for the unirradiated and irradiated have been offset by 0.01 absorbance units for presentation purposes. The spectra depicted are unaltered, except for the subtraction of air or "purge" bands. Each panel includes inserts for the 2400-2200 cm^{-1} and 1700-1000 cm^{-1} regions to show a more detailed view of the CO_2 and C_3O_2 bands and the AntCor photoproduct bands, respectively.

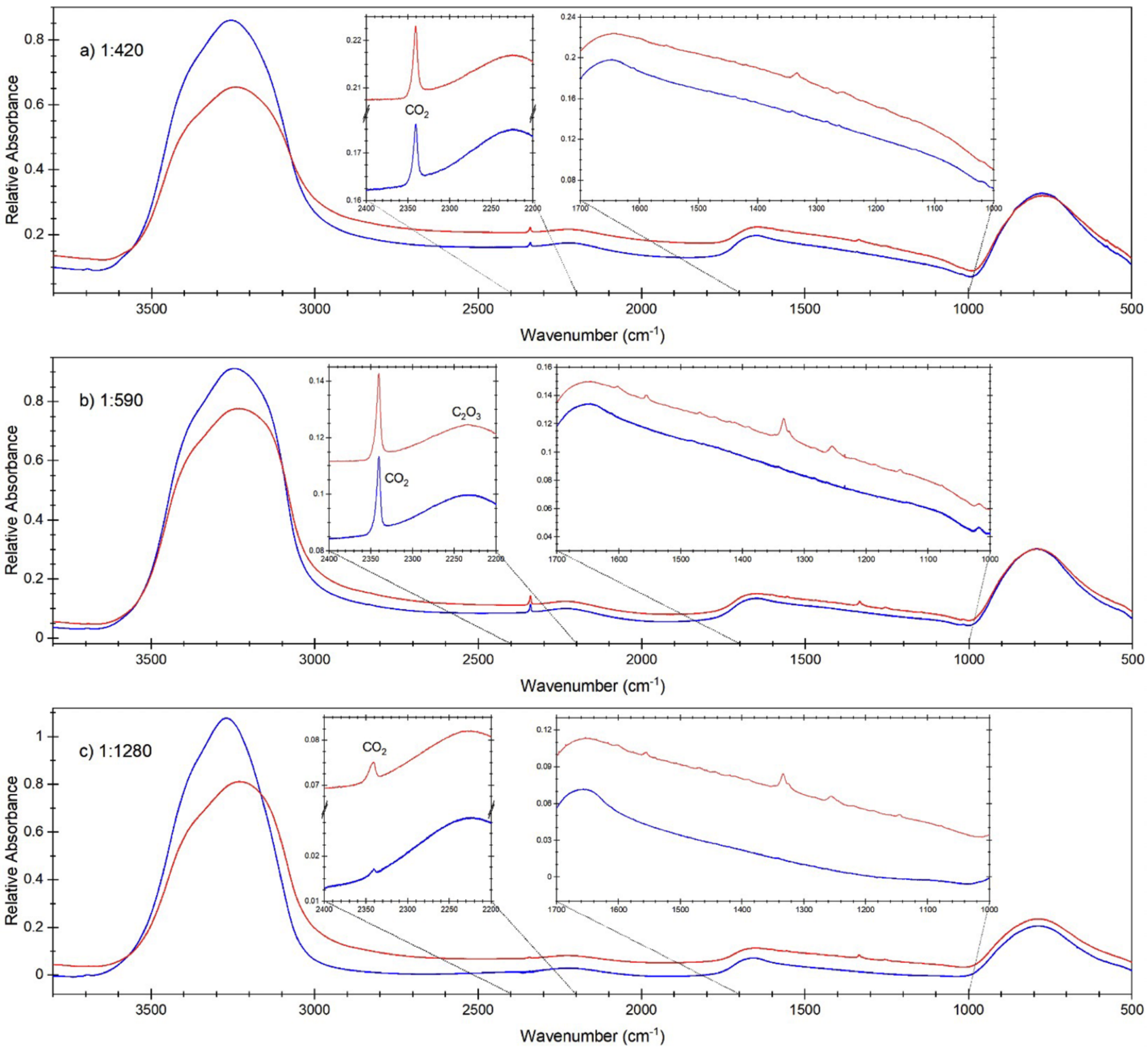

Figure S2: The full MIR spectra for unirradiated (blue) and irradiated (red) AntCor:H_2O samples, at concentrations a) 1:420, b) 1:590, c) 1:1280. The spectra for the unirradiated and irradiated have been offset by 0.01 absorbance units for presentation purposes. The spectra depicted are unaltered, except for the subtraction of air or "purge" bands. Each panel includes inserts for the 2400-2200 cm^{-1} and 1700-1000 cm^{-1} regions to show a more detailed view of the CO_2 and C_3O_2 bands and the AntCor photoproduct bands, respectively.
